# The juxtamembrane domain of StkP is phosphorylated and influences cell division in *Streptococcus pneumoniae*

**DOI:** 10.1128/mbio.03799-24

**Published:** 2025-04-08

**Authors:** Mélisse Hamidi, Sathya Narayanan Nagarajan, Vaishnavi Ravikumar, Virginie Gueguen-Chaignon, Cédric Laguri, Céline Freton, Ivan Mijakovic, Jean-Pierre Simorre, Stéphanie Ravaud, Christophe Grangeasse

**Affiliations:** 1 Molecular Microbiology and Structural Biochemistry, UMR 5086, Université Claude Bernard Lyon 1, CNRS 27098, Lyon, Auvergne-Rhône-Alpes, France; 2 Department of Biology and Biological Engineering, Chalmers University of Technology 11248, Gothenburg, Västra Götaland County, Sweden; 3 Protein Science Facility, CNRS UAR3444, INSERM US8, Université Claude Bernard Lyon 1, Ecole Normale Supérieur de Lyon 26911, Lyon, Auvergne-Rhône-Alpes, France; 4 Institut de Biologie Structurale, CEA, CNRS UMR 5075, Université Grenoble Alpes 27015, Grenoble, Auvergne-Rhône-Alpes, France; Institut Pasteur, Paris, France

**Keywords:** StkP, *Streptococcus pneumoniae*, protein kinase, bacterial cell division, juxtamembrane domain, StkP

## Abstract

Eukaryotic-like membrane Ser/Thr protein kinases play a pivotal role in different aspects of bacterial physiology. In contrast to the diversity of their extracellular domains, their cytoplasmic catalytic domains are highly conserved. However, the function of a long juxtamembrane domain (JMD), which connects the catalytic domain to the transmembrane helix, remains elusive. In this study, we investigated the function of the JMD of the Ser/Thr protein kinase StkP in the cell division of *Streptococcus pneumoniae*. We observed that the deletion of the JMD affected the ability of StkP to phosphorylate some of its endogenous substrates, thereby resulting in significant cell morphogenesis defects. Furthermore, multiple threonine residues were identified as being phosphorylated in the JMD. To investigate the functional significance of these phosphorylation sites, we conducted an integrative analysis, combining structural biology, proteomics, and bacterial cell imaging. Our results revealed that the phosphorylation of the JMD did not perturb the phosphorylation of StkP substrates. However, we observed that it modulated the timing of StkP localization to the division septum and the dynamics of cell constriction. We further demonstrated that phosphorylation of the JMD facilitated the recruitment of several cell division proteins, suggesting that it is required to assemble the division machinery at the division septum. In conclusion, this study demonstrates that the function of the JMD of StkP is modulated by phosphorylation and is critical for the cell division of *S. pneumoniae*. These observations may serve as a model for understanding the regulatory function of other bacterial Ser/Thr protein kinases.

**IMPORTANCE:**

How bacterial serine/threonine protein kinases are activated remains highly debated. In particular, models rely on the observations made with their eukaryotic counterparts, and only a few studies have investigated the molecular activation mechanism of bacterial serine/threonine protein kinases. This is particularly the case with regard to the juxtamembrane domain (JMD), which is proposed to contribute to kinase activation in numerous eukaryotic kinases. This study demonstrates that the juxtamembrane domain is likely not essential for the activation of the serine/threonine protein kinase StkP of *S. pneumoniae*. Rather, our findings reveal that it is required for cell division, where its phosphorylation affects the assembly of the division machinery at the division septum. These observations allow us to assign a function to the JMD in StkP-mediated regulation of pneumococcal cell division, thereby providing a new avenue for understanding the contribution of membrane serine/threonine protein kinases in the physiology of other bacteria.

## INTRODUCTION

Bacteria exhibit diverse cell morphologies, with rod-, spiral-, sphere-, and curved-cell shapes being particularly prevalent (1). To achieve these shapes, bacteria use a combination of both conserved and species-specific proteins and mechanisms to perform cell elongation, cell constriction, and ultimately cell separation. A critical step in these events is the assembly of the cell wall and in particular that of the peptidoglycan mesh (2). The latter consists of a giant polymer of glycan strands crosslinked by short peptides that encage the cell and confers its shape (3). Peptidoglycan assembly is achieved by different protein machineries at specific locations in the bacterial cell. The divisome, organized by the tubulin-like protein FtsZ (4), assembles peptidoglycan at the division site and drives cell constriction and separation. On the other hand, cell elongation can be achieved by inserting peptidoglycan either along the lateral side by the elongasome (5) or at the cell pole by the polarisome (6). The latter is organized by the tropomyosin-like protein DivIVA and assembles peptidoglycan at the cell pole (7), whereas the actin-like protein MreB shapes the elongasome that inserts peptidoglycan on the lateral side of the cell. However, some bacteria do not encode for one or more of these three cytoskeletal proteins. This is exemplified by numerous spherical and oblong bacteria (cocci and ovococci, respectively), which lack MreB (8, 9). Despite the presence of DivIVA in these bacteria, peptidoglycan is not synthesized at the cell pole, and it is only assembled at the mid-cell where it serves both for synthesizing the septal cross-wall and cell elongation (10).

One prominent model for studying cell division and cell morphogenesis in ovococci is the rugby-ball-shaped bacterium *Streptococcus pneumoniae* (11–13). Peptidoglycan assembly occurs exclusively at mid-cell and is orchestrated by FtsZ. Nevertheless, the analysis of the dynamics of several proteins of the divisome indicates that two distinct modes of peptidoglycan synthesis occur during cell septation (septal peptidoglycan synthesis) and elongation (peripheral peptidoglycan synthesis) (14). Both modes are required to achieve the pneumococcal ovoid shape. It is proposed that septal peptidoglycan synthesis is followed by its cleavage by hydrolases coupled to the insertion of peripheral peptidoglycan in the freshly cleaved region. This process leads to the formation of a lateral wall made of a composite of septal and peripheral peptidoglycan (13). Therefore, septal and peripheral syntheses should be finely regulated throughout the cell cycle of *S. pneumoniae*. In this regard, the serine/threonine kinase StkP has been demonstrated to play a critical role in regulating the morphogenesis of pneumococcus (15). This membrane serine/threonine kinase has been shown to phosphorylate various proteins, including those involved in cell division and morphogenesis. Notably, StkP phosphorylates the division site positioning protein MapZ (16, 17), the cell elongation regulator EloR (18, 19), the cell division protein DivIVA (20), and MacP, which regulates the class A PBP, PBP2a (19, 21). In addition, StkP also influences the function of other cell division proteins through direct protein-protein interaction. The localization of the class B PBP PBP2x at the division septum depends on its interaction with the extracellular domain of StkP (22). Furthermore, a recent study shows that StkP directs the function of the cell wall hydrolase LytB during the final cell separation of daughter cells (23, 24).

**Fig 1:**
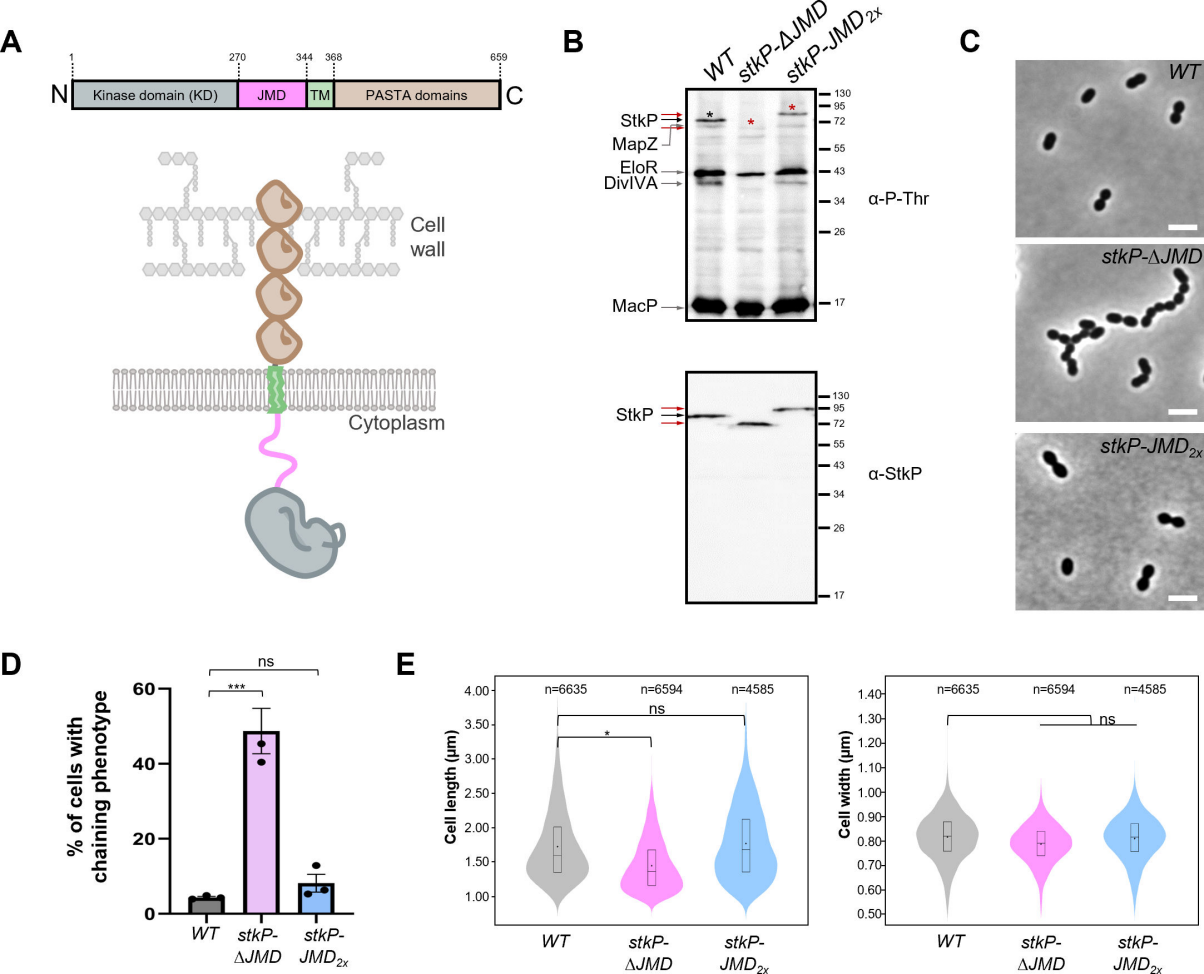
Role of the JMD in StkP activation and function is not required for StkP activation. (A) Schematic representation of StkP. The kinase domain (KD) is in gray, the juxtamembrane domain (JMD) in magenta, the transmembrane domain (TM) in light green, and the PASTA domains in brown. (B) Phosphorylation patterns. Western immunoblots of whole-cell lysates from WT, *stkP-*Δ*JMD,* and *stkP-JMD_2x_* cells. Whole-cell lysates were probed with anti-phosphothreonine antibodies (upper panel) or anti-StkP PASTA antibodies (lower panel). The black arrow shows WT StkP and the red arrows indicate StkP-ΔJMD and StkP-JMD_2x_. To help identify the corresponding autophosphorylation signals of StkP, black (WT StkP) and red (StkP-ΔJMD and StkP-JMD_2x_), asterisks were added above each band. The StkP substrates MapZ, DivIVA, EloR, and MacP are indicated by gray arrows. Images are representative of experiments made in triplicates. (C) Representative phase contrast microscopy images of WT, *stkP-*Δ*JMD,* and *stkP-JMD_2x_* cells. Scale bar, 2 µm. (D) Bar chart representing the percentage of pneumococcal cells harboring a chaining phenotype (minimum five cells per chain). Bars represent the mean (±SEM) of three independent experiments. The WT was compared separately with the *stkP-*Δ*JMD* or the *stkP-JMD_2x_* mutants with an unpaired t-test (ns, *P* > 0.05; ***P < 0.001). (E) Violin plots showing the distribution of the cell length (left panel) and cell width (right panel) for WT, *stkP-*Δ*JMD,* and *stkP-JMD_2x_* strains as determined using MicrobeJ. The distribution of the cell length and width are shown in gray for the WT strain, in magenta for the *stkP-*Δ*JMD* strain, and cyan for the *stkP-JMD_2x_* strain. The box indicates the 25th to the 75th percentile. The mean and the median are, respectively, indicated with a dot and a line in the box. Statistical comparison was done using the t-test (ns, *P* > 0.05; *P < 0.05). The *n* values represent the number of cells analyzed. Experiments were conducted in four replicates.

StkP is a membrane protein belonging to the Hanks-type kinase superfamily, which is commonly found in eukaryotes (15, 25). Homologs of StkP are also found in the bacterial phyla Firmicutes and Actinobacteria (26, 27). StkP comprises an extracellular domain and a cytoplasmic catalytic domain that is linked to a transmembrane helix via a long juxtamembrane domain (JMD) (Fig. 1A) (28). The structural organization of the cytoplasmic kinase domain is well-conserved among eukaryotic and bacterial serine/threonine kinases. However, the extracellular domain is not systematically conserved. In the case of StkP, the extracellular domain contains four PASTA domains (29), which bind peptidoglycan fragments and/or precursors (22, 30). It is proposed that StkP directs a signaling pathway dedicated to the coordination between peptidoglycan assembly and the division machinery (31). In this model, the function of the JMD remains elusive. In eukaryotic membrane kinases, the JMD has been reported to serve different functions, such as amplifying the activating signals to the kinase domain or autoinhibiting the catalytic domain activation (32–34). In addition, the length and sequence of the JMD vary among protein kinases, notably among StkP homologs, thus suggesting kinase and/or species-specific regulatory functions (28). In StkP and its close homologs such as PknB in *M. tuberculosis* and PrkC in *B. subtilis*, several phosphorylation sites have been identified within the JMD (35–37). However, their specific functional roles remain to be characterized.

In this study, we investigated the function of the JMD in StkP and its role in regulating pneumococcal cell division and morphogenesis. We identified all the phosphorylation sites within the JMD and revealed that this intrinsically disordered domain is essential for the phosphorylation of StkP substrates independently of its phosphorylation. In addition, we showed that the phosphorylation of the JMD influences the assembly of the division machinery at the division septum, thereby promoting cell constriction. Overall, this study offers the first molecular and structural insights into the role of the JMD in StkP and could serve as a model for other bacterial Hanks-type serine/threonine protein kinases.

## RESULTS

### The JMD is not required for StkP activation

To investigate the role of the juxtamembrane domain (JMD) of StkP, we constructed a strain in which StkP lacks this domain (*stkP-*Δ*JMD*) (Fig. 1A). The expression of *stkP-*∆*JMD* was confirmed by Western blot analysis (Fig. 1B). While the *stkP-*Δ*JMD* mutant exhibits no significant defect in cell growth (Fig. S1A), it displays an aberrant cell morphology (Fig. 1C). In particular, at least 40% of the cells were chained (Fig. 1D), indicative of impaired daughter cell final separation. Morphometric measurements further revealed that cell length, but not cell width, is significantly reduced compared to the wild-type (WT) strain (Fig. 1E). These defects were reminiscent of those detected for cells devoid of *stkP* (15). To determine whether the JMD deletion affects the kinase activity of StkP, we analyzed the *in vivo* phosphorylation pattern of the *stkP-*Δ*JMD* mutant (Fig. 1B). We detected that StkP retained a certain level of activity as indicated by its weak autophosphorylation signal, which is likely due to the phosphorylation of its activation loop (28). This autophosphorylation signal is also weaker compared to the WT strain due to the lack of phosphorylation within the JMD (see below). In addition, and importantly, the phosphorylation signal of MacP was comparable to that observed in the WT strain, confirming that StkP remained indeed active. However, the phosphorylation of the StkP substrates MapZ, EloR, and DivIVA (15, 16, 18) was severely impaired. This suggests that although StkP remains active without the JMD, its ability to phosphorylate some of its substrates depends on the presence of this domain. To ensure that the lack of phosphorylation of certain substrates was not due to the delocalization of StkP, we constructed an N-terminal GFP fusion of StkP lacking the JMD (*gfp-stkP-*Δ*JMD* strain) (Fig. S1B). This fusion was integrated at its native chromosomal loci and expressed under the control of the native promoter, serving as the sole source of StkP. As previously reported, StkP is a late cell division protein that localizes at the division septum (31). The GFP-StkP-ΔJMD fusion is stable (Fig. S1C) and localizes correctly to the division septum, similar to that observed for GFP-StkP (Fig. S1B) (15). This confirms that the substrate phosphorylation defects are not a consequence of StkP delocalization. The JMD is therefore not required for the septal localization of StkP but critical for the phosphorylation of some substrates while having no impact on the phosphorylation of others.

The JMD is predicted to be intrinsically disordered in bacterial serine/threonine protein kinases (36) and see below). Thus, we reasoned that the JMD could serve to bring flexibility between the transmembrane helix to allow the catalytic kinase domain to phosphorylate some of its substrates. To test this hypothesis, we constructed another strain in which we duplicated the JMD in tandem (*stkP-JMD_2x_*). Surprisingly, the phosphorylation pattern (Fig. 1B), the growth (Fig. S1A) as well as the morphology of *stkP-JMD_2x_* cells are indistinguishable from that of WT cells (Fig. 1C through E). Altogether, our observations show that the JMD is dispensable for the activation of StkP and that the precise length of the JMD is not critical for StkP activation. Furthermore, these observations suggest that the JMD may facilitate the positioning and interaction of the catalytic domain to position and interact with its phosphorylation substrates.

### Phosphorylation of 7 threonine residues within the JMD affects pneumococcal cell morphology

**Fig 2:**
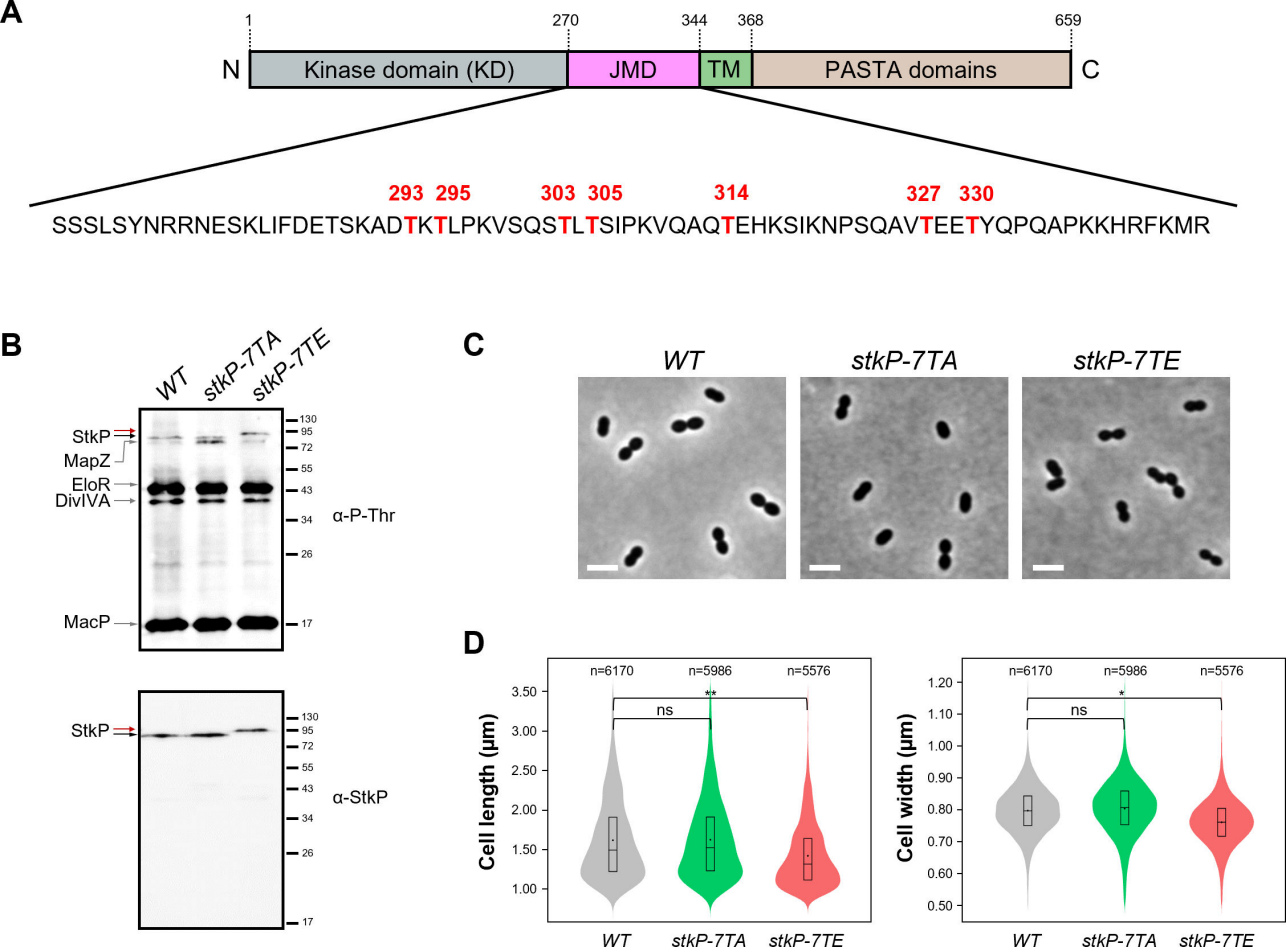
Phosphorylation of the JMD and pneumococcal cell morphology. (A) The amino acid sequence of the juxtamembrane domain (JMD) is shown to highlight the seven phosphorylation sites (T293, T295, T303, T305, T314, T327, and T330) identified by mass spectrometry (see Fig. S2 and S3). (B) Phosphorylation patterns. Western immunoblots of whole-cell lysates from WT, *stkP-7TA,* and *stkP-7TE* cells. Whole-cell lysates were probed with anti-phosphothreonine antibodies (upper panel) or anti-StkP PASTA antibodies (lower panel). The black arrow shows WT StkP and the red arrows indicate StkP-7TA and StkP-7TE. The StkP substrates MapZ, DivIVA, EloR, and MacP are indicated by gray arrows. Images are representative of experiments made in triplicates. (C) Representative phase contrast microscopy images of WT, *stkP-7TA,* and *stkP-7TE* cells. Scale bar, 2 µm. (D) Violin plot showing the distribution of the cell length (left panel) and cell width (right panel) for WT, *stkP-7TA,* and *stkP-7TE* (red) strains as determined using MicrobeJ. The distribution of the cell length and width is shown in gray for the WT strain, in green for the *stkP-7TA* strain, and red for the *stkP-7TE* strain. The box indicates the 25th to the 75th percentiles. The mean and the median are respectively indicated with a dot and a line in the box. Statistical comparison was done using the *t*-test (ns, *P* > 0.05; *P < 0.05; **P < 0.01). The *n* values represent the number of cells analyzed. Experiments were performed in four replicates.

Previous studies have reported that some residues of the JMD of bacterial serine/threonine protein kinases can be phosphorylated, in particular PknB from *M. tuberculosis* and PrkC from *B. subtilis* (36, 37). A phosphoproteome of *S. pneumoniae* was recently reported, identifying four threonines (293, 295, 305, and 330) as being phosphorylated in the JMD of StkP when cells were grown in the presence of ampicillin (35). Indeed, autophosphorylation of StkP as well as the phosphorylation of StkP endogenous substrates was shown to increase *in vivo* when cells were grown in the presence of a sublethal concentration of ampicillin (38). To get a comprehensive characterization of all the phosphothreonines within the JMD of StkP, we purified GFP-StkP from pneumococcal cells grown in the absence or presence of ampicillin and performed a phosphoproteomic analysis after a phosphopeptide enrichment procedure. As shown in Fig. S2A through E, threonines 293, 295, 303, 314, and 330 are phosphorylated when cells are grown in the absence of ampicillin. The same analysis performed with cells grown in the presence of ampicillin allowed us to detect the phosphorylation of threonines 293, 303, 305, 327, and 330 (Fig. S3A through E). Together, and combined with the previous phosphoproteomic study (35), seven threonines were found to be phosphorylatable in the JMD of StkP (Fig. 2A).

To investigate the influence of the JMD phosphorylation on pneumococcal cell growth and morphology, two mutants were constructed in which the aforementioned seven threonines were substituted with either alanine or glutamic acids, generating thus two constructs, designated *stkP-7TA* and *stkP-7TE*, which mimicked either permanent dephosphorylation of StkP (phosphoablative mutant) or permanent phosphorylation of StkP (phosphomimetic mutant), respectively. Subsequently, the growth and viability of the two mutant strains were analyzed, and no significant difference was observed between them and the WT strain (Fig. S4A and B). Similarly, the phosphorylation patterns exhibited no significant differences (Fig. 2B). However, careful inspection of cell morphology revealed notable differences between the phosphomimetic and the phosphoablative mutants of StkP (Fig. 2C). While the *stkP-7TA* cells were undistinguishable from WT cells, the *stkP-7TE* mutant exhibited significantly shorter and thinner cells in comparison to the WT cells (Fig. 2D). Morphometric measurements revealed that the average cell length and width were 1.43 ± 0.4 µm and 0.76 ± 0.08 µm, respectively, for *stkP-7TE* cells. In comparison, the calculated values for WT cells were 1.62 ± 0.5 µm and 0.8 ± 0.08 µm. This observation was accompanied by an increased proportion of cells with reduced length and width. In particular, 39% of the *stkP-7TE* cells exhibited a cell length below the first quartile (Q1), compared to 25% for WT cells, and 40% had a width below Q1, compared to 25% in the WT (Fig. 2D). However, no cell chaining was detected for both mutants (Fig. S4C). These findings demonstrate that the dephosphorylation of the JMD does not alter cell division. By contrast, mutations mimicking permanent phosphorylation of the JMD affect the morphology of pneumococcal cells but not their final separation as observed when the JMD is deleted (Fig. 1D).

### Localization of StkP and FtsZ occurs at the division septum at an early stage in the *stkP-7TE* mutant

**Fig 3:**
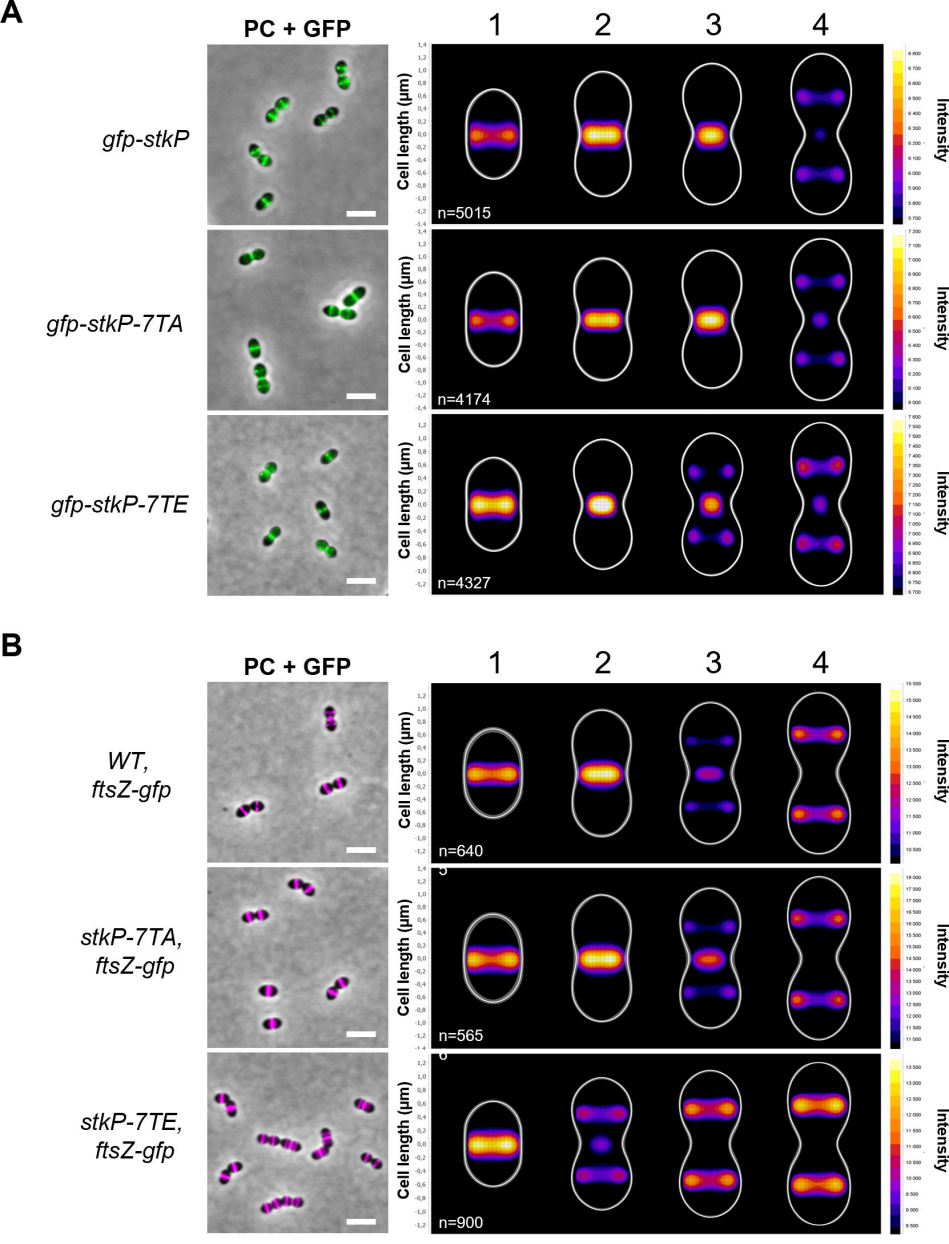
Localization of GFP-StkP and FtsZ-GFP in WT, *stkP-7TA,* and *stkP-7TE* strains. (A) The left panel shows overlays between phase-contrast and GFP images of *gfp-stkP, gfp-stkP-7TA,* and *gfp-stkP-7TE* cells, respectively. Scale bar, 2 µm. The heatmap (right panel) represents the localization patterns of GFP-StkP, GFP-StkP-7TA, and GFP-StkP-7TE during the cell cycle. Four stages of cell division are represented (1, 2, 3, and 4). The *n* values represent the number of cells analyzed. Experiments were performed in triplicate. (B) The left panel shows overlays between phase-contrast and GFP images for WT, *ftsZ-gfp; stkP-7TA, ftsZ-gfp* and *stkP-7TE, ftsZ-gfp* cells. Scale bar, 2 µm. The heatmap (right panel) displays the localization of FtsZ-GFP in WT, *stkP-7TA,* and *stkP-7TE* cells during the cell cycle. Four stages of cell division are represented (1, 2, 3, and 4). The *n* values represent the number of cells analyzed. Experiments were performed in triplicate.

We hypothesized that the cell shape defects observed for the *stkP-7TE* mutant could be attributed to the aberrant localization of StkP. To study the dynamics of StkP during the cell cycle, we constructed an N-terminal GFP fusion of the two phosphorylation mutants of the JMD (*gfp-stkP-7TA* and *gfp-stkP-7TE*). We first verified the stability of the fusions (Fig. S5A). The cells expressing these two constructs grew similarly to *gfp-stkP* cells, and the morphogenesis defects observed for the phosphomimetic mutant were preserved, indicating that both fusions were stable and fully functional (Fig. S5C and D). The analysis of StkP dynamics during the cell cycle showed that GFP-StkP remains localized at the division septum throughout the entire cell cycle in WT cells. More precisely, StkP relocates to the cell equator, which corresponds to the future division site of the daughter cells, only at the very late stage of the cell cycle (Fig. 3A) (39). The localization pattern observed in cells producing the phosphoablative GFP-StkP-7TA was similar. By contrast, the phosphomimetic mutant displayed an early localization of GFP-StkP-7TE at the future division site, with a fluorescence signal at the cellular equator as early as in stage 3 (Fig. 3A). These results indicate that JMD phosphorylation influences StkP dynamics throughout the cell cycle, suggesting that the activity of the division machinery could be modulated by JMD phosphorylation of StkP.

To verify this hypothesis, we selected FtsZ, a key protein of the division machinery (40, 41), as a proxy to track the assembly of the division machinery during the cell cycle. We thus constructed *stkP-7TA* and *stkP-7TE* strains producing the functional FtsZ-GFP fusion (*stkP-7TA, ftsZ-gfp* and *stkP-7TE, ftsZ-gfp*). The FtsZ-GFP fusions were stable in these two genetic backgrounds (Fig. S5B) and cell growth of *stkP-7TA, ftsZ-gfp* and *stkP-7TE, ftsZ-gfp* strains was comparable to that of WT, *ftsZ-gfp strain* with only a slight delay in lysis (Fig. S5E). Similarly, the morphogenesis defects observed for the phosphomimetic mutant *stkP-7TE* were also conserved in the *stkP-7TE, FtsZ-GFP* strain (Fig. S5F). As previously described (31), the analysis of FtsZ dynamics in WT revealed that FtsZ-GFP localized at the division septum in the early and intermediate stages (1, 2, and 3) of cell division (Fig. 3B). At stage 3, FtsZ-GFP relocates to the cellular equator, while it still persists at the septum before being completely relocated at stage 4. FtsZ-GFP dynamics in the *stkP-7TA* strain was similar to that observed in the WT strain. However, an obvious early localization of FtsZ-GFP was observed in the phosphomimetic strain with a fluorescence signal detected at the cell equator as soon as stage 2 (Fig. 3B). In conclusion, these observations indicate that JMD phosphorylation significantly impacts the timing of the localization of the cell division machinery at the division septum. It may also be suggested that the reduction in cell length observed in the *stkP-7TE* mutant is a consequence of the early localization of the divisome.

### The phosphomimetic form of StkP promotes cell constriction

**Fig 4:**
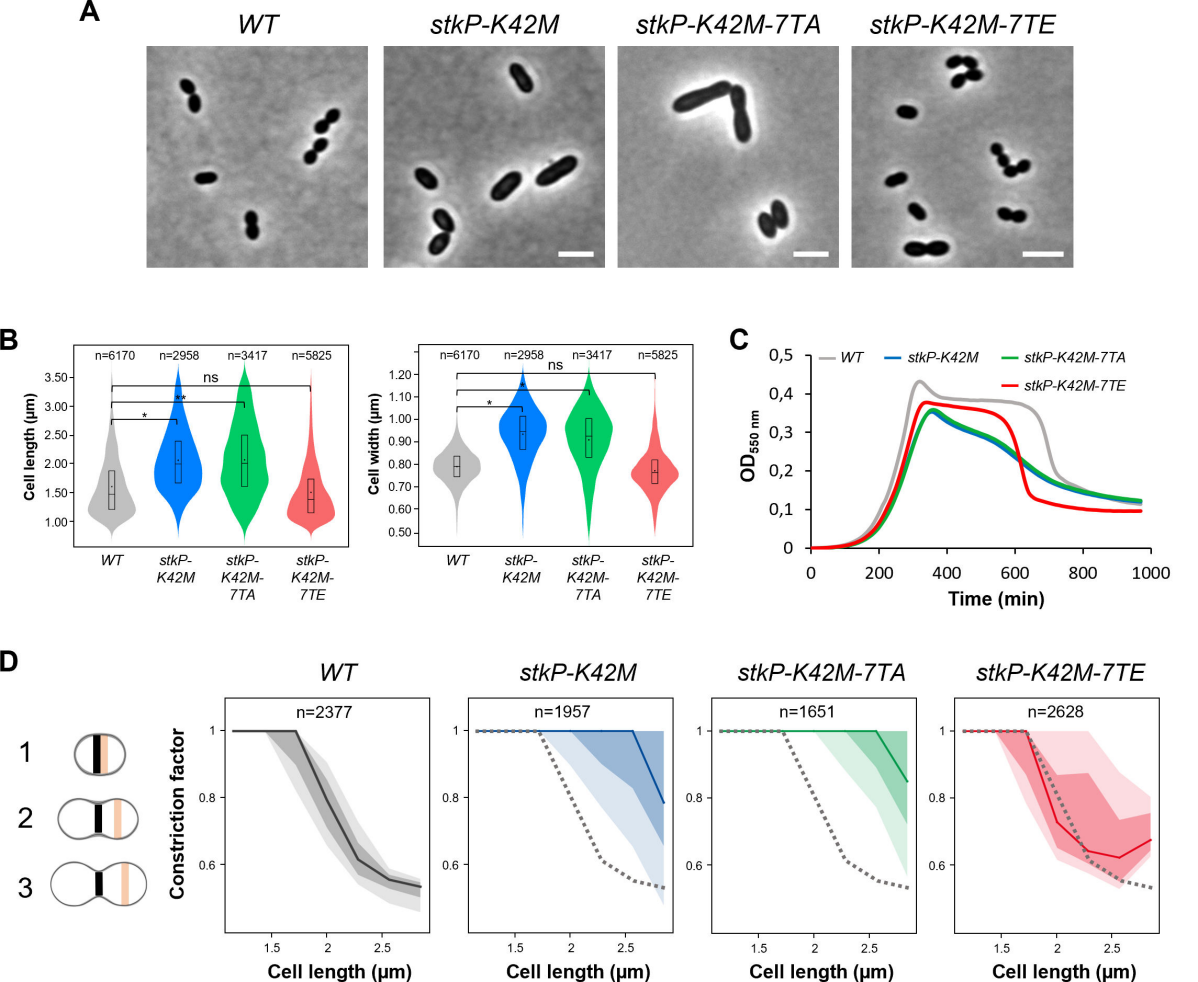
Impact of phosphomimetic and phosphoablative mutations on the cell shape of the *stkP-K42M* strain. (A) Representative phase contrast microscopy images of WT, *stkP-K42M*, *stkP-K42M-7TA,* and *stkP-K42M-7TE* cells. Scale bar, 2 µm. (B) Violin plot showing the distribution of the cell length (left panel) and cell width (right panel) for WT (gray), *stkP-K42M* (blue), *stkP-K42M-7TA* (green), and *stkP-K42M-7TE* (red) strains as determined using MicrobeJ. The box indicates the 25th to the 75th percentiles. The mean and the median are respectively indicated with a dot and a line in the box. Statistical comparison was done using the *t*-test (ns, *P* > 0.05; *P < 0.05; and **P < 0.01). The *n* values represent the number of cells analyzed. Experiments were performed in triplicate. (C) Growth of WT, *stkP-K42M*, *stkP-K42M-7TA,* and *stkP-K42M-7TE* strains. Experiments were performed in triplicate. (D) Dispersion of the constriction factors as a function of the cell length in WT (gray), *stkP-K42M* (blue), *stkP-K42M-7TA* (green), and *stkP-K42M-7TE* (red) strains. Three stages of cell division are illustrated (left panel). The constriction factor (CF) is calculated as the ratio of cell width at the division septum (CellWidth_sep_, shown in black) to cell width at the cell equator (CellWidth_equ_, shown in orange). For each strain, the solid line represents the median, the dark-shaded area indicates the interquartile range (Q1–Q3) and the light-shaded area represents the amplitude (min–max) (right panels). The dashed line indicates the WT median for comparison with mutants. The *n* values represent the number of cells analyzed in a single representative experiment. Experiments were performed in triplicate.

Since our observations suggest that JMD phosphorylation decreases the cell length and promotes the early localization of the division machinery at the division septum (Fig. 3B), we hypothesized that the *stkP-7TE* mutation may trigger early cell constriction. To test this hypothesis, we generated phosphorylation mutants of the JMD of StkP in a cellular context where cell constriction is hindered, resulting in cell elongation. Previous studies have shown that the K42M mutation in StkP, which affects the catalytic lysine (K42) and abolishes its kinase activity, leads to cell elongation with the presence of multiple non-constricted septa (15). We thus introduced the phosphoablative or phosphomimetic mutation of the JMD in the *stkp-K42M* genetic background and analyzed the cell shape of *stkp-K42M-7TA* and *stkp-K42M-7TE* cells to determine whether the JMD phosphorylation is sufficient to promote cell constriction, even in the absence of functional kinase activity. We checked that the generated mutants were stable and that their phosphorylation profiles showed a complete loss of StkP kinase activity, as expected with the K42M mutation (Fig. S6). Under the microscope, *stkP-K42M-7TA* cells displayed a morphology similar to *stkP-K42M* cells, both being significantly longer and wider than WT cells (Fig. 4A and B). By contrast, *stkP-K42M-7TE* cells did not exhibit major morphogenetic defects and closely resembled WT cells (Fig. 4A). Specifically, the average cell length and width are 2.06 ± 0.61 µm and 0.91 ± 0.13 µm for *stkP-K42M-7TA* cells, compared to 1.49 ± 0.48 µm and 0.77 ± 0.1 µm for *stkP-K42M-7TE* cells (Fig. 4B). This result was further supported by cell growth analyses. While the phosphoablative *stkP-K42M-7TA* mutant showed severely impaired cell growth, characterized by early lysis similar to that of the *stkP-K42M* mutant, the phosphomimetic *stkp-K42M-7TE* mutant exhibited improved cell growth (Fig. 4C). These observations indicate that the phosphorylation of the JMD compensates for the loss of StkP activity, rendering the kinase activity of StkP dispensable for normal cell morphogenesis and growth. They also support our hypothesis that the expression of JMD-7TE facilitates the assembly of the division machinery and promotes cell constriction.

To analyze in detail the impact of JMD phosphorylation on cell constriction, we examined the progression of cell constriction during the cell cycle. This involved calculating a constriction factor (CF), based on the ratio between the cell width at the division septum (CellWidth_sep_) and the cell width at the cell equator (CellWidth_equ_), (CF =$\frac{Cell\;width_{sep}}{Cell\;width_{equ}}$) (Fig. 4D). Consequently, in newborn WT cells (stage 1), the CellWidth_sep_ and CellWidth_equ_ are equal, resulting in a constriction factor of CF = 1. As cell elongation and cell constriction progress (stages 2 and 3), the width at the division septum decreases, resulting in a CF <1, which indicates that the cell is undergoing constriction. Stage 3 of division corresponds to cells that have nearly completed constriction and are about to separate, resulting in two daughter cells. The representations of CFs of WT, *stkP-K42M, stkP-K42M-7TA,* and *stkP-K42M-7TE* cells revealed that the phosphomimetic mutant exhibited a constriction profile comparable to that of WT, though with increased variability. By contrast, both *stkP-K42M* and *stkP-K42M-7TA* mutants showed a blockage in cell constriction (Fig. 4D). These findings again demonstrate that the 7TE mutation alleviates the constriction block of the *stkP-K42M* mutant, thereby highlighting the critical role of JMD phosphorylation in controlling cell constriction. Remarkably, phosphorylation of the JMD alone is sufficient to initiate the cell constriction process, even in the absence of StkP substrate phosphorylation (Fig. S6).

### Both phosphomimetic and phosphoablative forms of the JMD are intrinsically disordered

**Fig 5:**
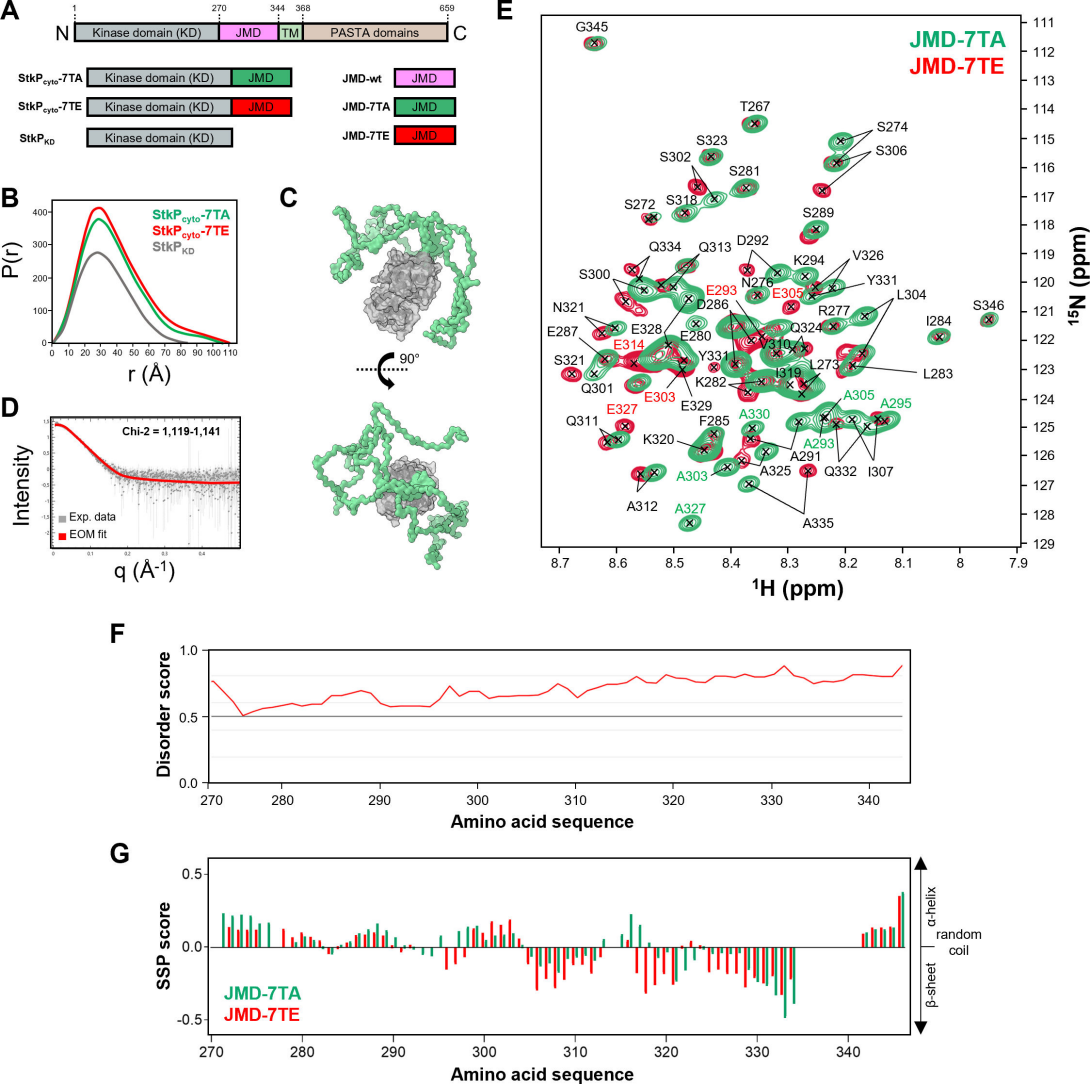
Structural analysis of the JMD of StkP. (A) Constructs of StkP used for the structural analyses: the cytoplasmic domain in its phosphoablative (StkP_cyto_-7TA, in gray/green) or phosphomimetic (StkP_cyto_-7TE, in gray/red) forms, the kinase domain (StkP_KD_ in gray) and the juxtamembrane domain in its WT (JMD-wt, in magenta), phosphoablative (JMD-7TA, in green) and phosphomimetic (JMD-7TE, in red) forms. (B) SAXS pair-distance distribution for StkP_KD_ (gray), StkP_cyto_-7TA (green), and StkP_cyto_-7TE (red). (C) StkP_cyto_-7TA ensemble refined by EOM (Ensemble Optimization Method). A chain of dummy residues for the juxtamembrane domain (JMD, in green) and the Alphafold prediction structure of the kinase domain (KD, gray) were used. Five JMD conformers best representing the experimental scattering profile are depicted. (D) SAXS EOM fit. The statistical analyses of the fit were carried out using the reduced Chi-2 method (one-tailed distribution). Several models were refined by EOM with a Chi-2 value between 1,118 and 1,141. (E) Overlay of the ^1^H–^15^N BEST-TROSY spectra recorded for ^13^C,^15^N-labeled JMD-7TA (green) and JMD-7TE (red). Protein samples were prepared in 25 mM in Bis-Tris (pH 7.4), and 150 mM NaCl buffer. Assignments of the resonances of JMD-7TA and JMD-7TE are reported in black, and the mutated residues 293, 295, 303, 305, 314, 327, and 330 are indicated in green and red for phosphoablative (JMD-7TA) and phosphomimetic (JMD-7TE) constructs, respectively. Only minimal chemical shift differences are observed between these spectra. (F) Disorder scores were predicted along the protein sequence of the juxtamembrane domain (JMD) of StkP by the IUPred software (http://iupred2a.elte.hu/). Highly disordered regions correspond to scores above 0.5. (G) Secondary structure propensity (SSP) scores were calculated from C_α_ and C_β_ NMR chemical shifts of JMD-7TA (in green) and JMD-7TE (in red). Positive values represent α-helices propensity and negative values represent β-sheets propensity.

Next, we aimed to elucidate the molecular mechanisms underlying the promotion of cell constriction by phosphomimetic mutations. To address this, we hypothesized that phosphorylation may influence the structural organization of the JMD and/or its positioning relative to the kinase domain, as observed for eukaryotic kinases (42–45). However, no experimental structure has been determined for either the JMD or the kinase domain of StkP. Thus, to address the current lack of structural and dynamic information about the JMD, we first used SAXS to study the solution structure of the cytoplasmic domain (comprising the JMD and the catalytic domain) of StkP. We first overproduced and purified to homogeneity the catalytic domain (residues 1–287: StkP_KD_) and the cytoplasmic domain of StkP in its phosphoablative and phosphomimetic mutant forms (residues 1–344: StkP_cyto_-7TA and StkP_cyto_-7TE) (Fig. 5A; Fig. S7A). The distance distribution functions P(r) calculated from the scattering data for the StkP_KD_ sample displayed a symmetric bell-shaped curve characteristic of a structured globular domain (Fig. 5B). The more elongated and asymmetric shape of the P(r) functions calculated for the StkP_cyto_-7TA and StkP_cyto_-7TE samples corroborates the disordered and flexible nature of the JMD (Fig. 5B and see below). We next compared the radius of gyration (Rg) and maximum intermolecular distance (Dmax) calculated for StkP_KD_, StkP_cyto_-7TA, and StkP_cyto_-7TE (Table S1). The presence of the JMD in StkP_cyto_-7TA and StkP_cyto_-7TE increased the estimated Rg (respectively 31.66 and 30.86 Å) and Dmax (respectively 113 and 111.5 Å) compared to StkP_KD_ (Rg = 26.04 Å; Dmax = 87 Å), suggesting that the JMD adopts an extended state relative to the kinase domain (Table S1). In addition, since the Rg and Dmax values were very similar regardless of the mutation of the seven threonine residues in the JMD, our results demonstrate that the phosphorylation state of the JMD does not significantly alter its global conformation. The ensemble optimization method (EOM) was also used to model the solution structure of the cytoplasmic domain of StkP (Fig. 5C). The catalytic domain was treated as a rigid body and the flexible JMD as a random chain within Ramachandran constraints. The selected conformers that fitted the experimental scattering curve with Chi-2 values comprised between 1.119 and 1.141 illustrated the significant flexibility of the JMD (Fig. 5C and D).

To further investigate the molecular structure and dynamics of the JMD, we performed liquid-state NMR experiments. We overproduced and purified to homogeneity the JMD domain in its WT, phosphoablative, and phosphomimetic mutated forms (residues 270–344: JMD-wt, JMD-7TA, and JMD-7TE), and the cytoplasmic domain of StkP in its phosphoablative and phosphomimetic mutated forms (residues 1–344: StkP_cyto_-7TA and StkP_cyto_-7TE). These proteins were labeled with ^13^C and/or ^15^N (Fig. 5A; Fig. S7B). The ^1^H-^15^N correlation spectra of JMD-wt, JMD-7TA, and JMD-7TE displayed intense, narrow peaks with very low chemical shift dispersion, indicative of a disordered region (Fig. S7C). Furthermore, overlaying the ^1^H-^15^N BEST-TROSY spectra of StkP-7TE with JMD-7TE and StkP-7TA with JMD-7TA indicated that the JMD does not interact with the kinase domain and that its presence does not influence the JMD conformation (Fig. S7D). This result rules out the hypothesis of a phosphorylation-mediated interaction between the JMD and the kinase domain, as is observed in some eukaryotic protein kinases (42, 46).

Next, we assigned backbone resonances for the JMD-7TA and JMD-7TE samples using the standard set of 3D ^1^H,^13^C, and ^15^N multidimensional NMR experiments (Fig. 5E). The backbone resonance assignment was over 80% complete for JMD-7TA and nearly 70% for JMD-7TE, enabling the calculation of the residue-specific secondary structure propensities (SSP) for most residues (47) (Fig. 5G). Resulting SSP scores were then deduced based on assigned ^13^C (C_α_ and C_β_) chemical shifts for each residue assigned along the protein sequence, with values ranging between −1 (fully elongated/β-strand structure) and 1 (fully formed helix) (Fig. 5G). The data indicated that the JMD remains predominantly disordered, as predicted (Fig. 5F) in both phosphorylation states, though JMD phosphorylation slightly affects its propensity to form local secondary structures. Overall, our results confirmed that the JMD of StkP is intrinsically disordered and that its phosphorylation does not significantly alter either its conformation or positioning relative to the kinase domain.

### The phosphomimetic form of the JMD favors the recruitment of proteins from the cell division machinery

**Fig 6:**
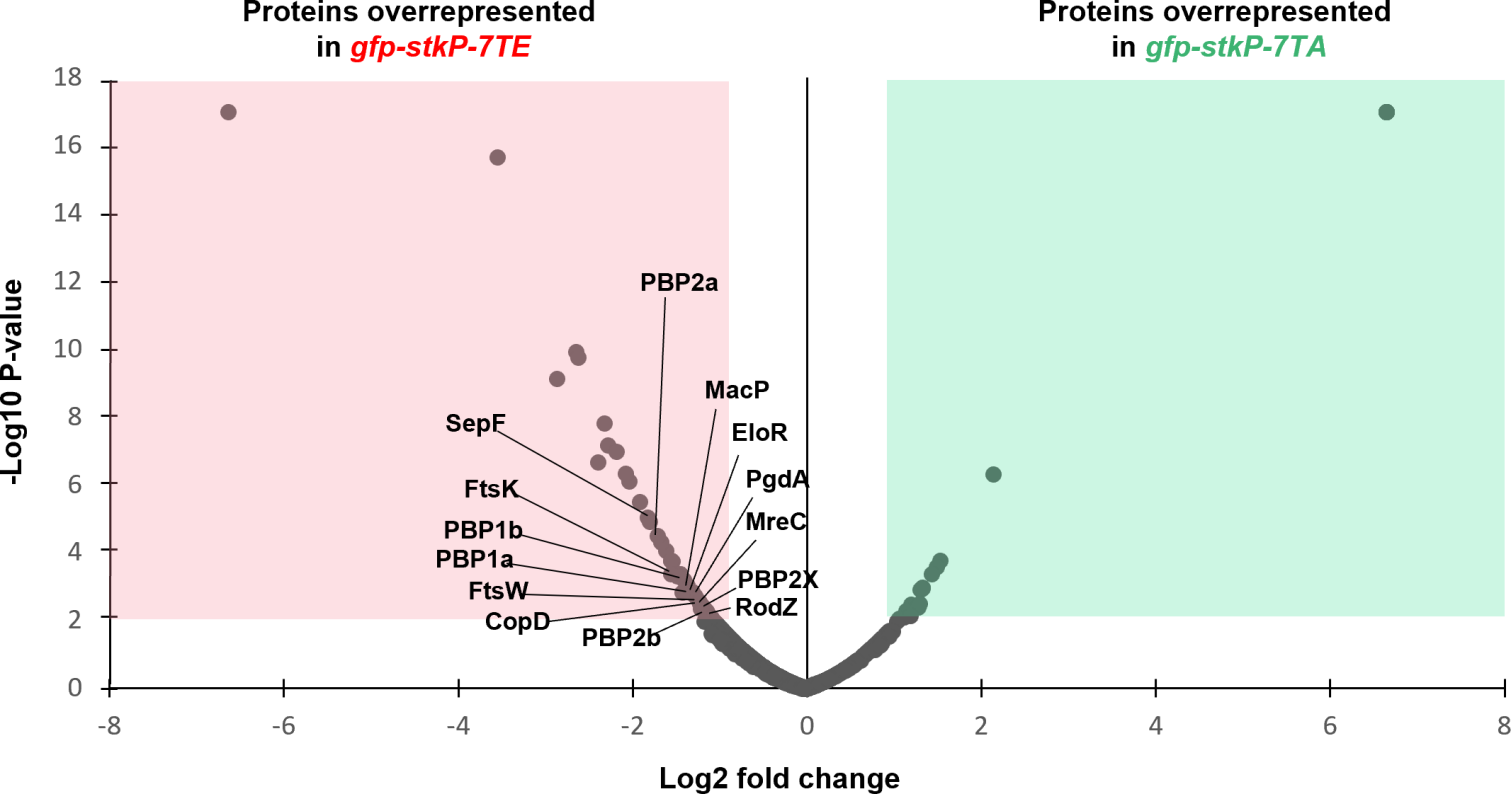
Proteomic analysis of GFP-StkP-7TA and GFP-StkP-7TE partners. The volcano plot shows proteins co-immunoprecipitated and overrepresented in the *gfp-stkP-7TA* (green area) and *gfp-stkP-7TE* (red area) strains. Data correspond to the average of three biological replicates. Proteins are significantly overrepresented in the GFP-StkP-7TE sample when log2 (fold change) < −0.85 and –log10 (*P*-value) > 2. Proteins are significantly overrepresented in the GFP-StkP-7TA sample when log2 (fold change) > 0.85 and –log10 (*P*-value) > 2.

We hypothesized that JMD phosphorylation may influence StkP’s interactions with other proteins involved in cell division. Indeed, this property has been observed for eukaryotic receptor tyrosine kinases where JMD phosphorylation serves as a recruitment site for downstream signaling proteins (44, 46, 48). To explore this possibility, we employed a quantitative label-free mass spectrometry approach to analyze the relative amounts of proteins immunoprecipitated with StkP in the *gfp-stkP-7TA* and *gfp-stkP-7TE* strains. Approximately 1,000 proteins were detected in both samples, with 24 proteins significantly overrepresented in the GFP-StkP-7TA sample and 79 in the GFP-StkP-7TE sample (Table S2) (Fig. 6). No cell division proteins were identified among those overrepresented in the GFP-StkP-7TA sample. Instead, the enriched proteins were primarily involved in metabolic processes, such as the adenylosuccinate lyase PurB, the galactokinase GalK, or the riboflavin biosynthesis protein RibD (Table S2).

By contrast, the GFP-StkP-7TE sample showed 14 proteins, among the 79 overrepresented proteins, that are well known for their roles in cell division, particularly in peptidoglycan synthesis (Fig. 6; Table S2). These include notably all the penicillin-binding proteins (PBP1a, PBP2a, PBP1b, PBP2b, and PBP2x) encoded in *S. pneumoniae* that polymerize and/or cross-link the peptidoglycan network (49). In addition, the peptidoglycan-modifying protein PgdA was also detected (50). The well-characterized cell division proteins FtsW, MreC, and RodZ, which assist and organize peptidoglycan assembly (51–53), were enriched together with proteins involved in the assembly of the division machinery (SepF) and the coordination between cell division and chromosome segregation (FtsK) (54, 55). Last, several regulatory proteins, like the recently identified spatiotemporal regulator of PBP1a and PBP2b, CopD (56), the regulator of cell elongation EloR (18), and the PBP2a regulator MacP (21) were also detected. Interestingly, MacP and EloR are phosphorylated by StkP (19, 21). Besides these cell division proteins, several proteins that may have roles related to cell division were also enriched, although primarily involved in other processes. For instance, the ferric transporter FatB was recently reported to be downregulated in response to cell wall stress, and the regulatory protein GlnR affects the pneumococcal cell wall physiology when co-inactivated with the regulator CodY (35, 57). Another notable protein, Spr0889, that is homologous to PopT (58) was recently shown to be implicated in the recycling of undecaprenyl monophosphate, the lipid carrier of the peptidoglycan precursor, and teichoic acids in *S. pneumoniae* (59). In addition, the Psr protein, which is involved in teichoic acid synthesis, and the VicR protein, which is a component of the VicRK two-component system, whose regulon comprises several genes involved in cell wall biosynthesis, were also identified (60, 61). The other overrepresented proteins involved in metabolism, ribosome assembly, or membrane homeostasis processes are listed in Table S2. These results suggest that JMD phosphorylation of StkP promotes the recruitment of several key cell division proteins, highlighting its role in coordinating and/or scaffolding the assembly of the division machinery.

## DISCUSSION

In this study, we provide the first insights into the function of the juxtamembrane domain (JMD) of the serine/threonine kinase StkP of *S. pneumoniae,* and more generally, of a bacterial serine/threonine kinase. Our observations allowed us to show that the JMD is not an indispensable component for StkP autophosphorylation. However, it is required for the final separation of daughter cells and the phosphorylation of most of its endogenous substrates. We also showed that the JMD is phosphorylated on seven threonines *in vivo,* and we demonstrated that phosphorylation influences the assembly of the divisome and cell constriction but not final cell separation.

The JMD, which connects the transmembrane helix to the catalytic domain, varies in length or amino acid sequence across StkP bacterial homologs (28). In previous studies, the deletion of the JMD has a negligible impact on the *in vitro* catalytic activity of the two well-studied StkP homologs, PknB from *Mycobacterium tuberculosis* (36) and PrkC from *Bacillus subtilis* (37). However, the effect of JMD deletion on the *in vivo* activity of PknB and PrkC is yet to be investigated. The observation that StkP is still able to autophosphorylate, most probably on the two conserved threonine residues of its activation loop (28), suggests that deleting the JMD does not disrupt completely its kinase activity. In numerous eukaryotic membrane protein kinases, including receptor tyrosine kinases, cytokine receptors, immune receptors, and cell adhesion receptors, the JMD has been demonstrated to facilitate the transduction of signals sensed by the extracellular ligand-binding domains and kinase activation (42–45). More precisely, it would relay and/or amplify the signal from the transmembrane helix, which undergoes different motions, such as translation and rotation, upon signal sensing (62). This function is unlikely in StkP because duplicating the JMD between the transmembrane and catalytic domains does not change kinase activity or substrate phosphorylation (Fig. 1B). Indeed, given the flexibility of the JMD (Fig. 5) (36), it seems improbable that a motion of the transmembrane domain could be transmitted to the catalytic domain. In some eukaryotic protein kinases, the JMD has been also proposed to make contacts with the catalytic domain resulting in autoinhibition. As seen in the ephrin receptor EPBH2, this interaction is destabilized following ligand binding and subsequent phosphorylation of the JMD resulting in kinase activation (33, 46). In other eukaryotic protein kinases, such as the muscle-specific kinase (MUSK), the JMD autoinhibitory effect is mediated by a different mechanism (63). The JMD does not interact with the catalytic domain but interacts instead with another cytoplasmic protein to constrain the kinase domain. This has been demonstrated with the type-I transforming growth factor β (TGFβ) whose JMD inhibitory effect is suppressed upon interaction with the immunophilin FKB12 (64). In the case of StkP, our structural analysis (Fig. 5; Fig. S7D) revealed no interaction between its JMD and catalytic domain, and JMD phosphorylation does not significantly impact substrate phosphorylation (Fig. 2B). These findings strengthen our hypothesis that the JMD of StkP and its phosphorylation are likely not essential for StkP autophosphorylation. Given that StkP retains the ability to phosphorylate its substrates even when the JMD is duplicated, an alternative hypothesis is that the JMD may play a role in providing flexibility to the catalytic domain, facilitating optimal positioning for interaction with its endogenous substrates.

We identified seven phosphothreonines within the JMD of StkP (Fig. 2A) surpassing a previous global phosphoproteomic study that detected only four of these phosphothreonines (35). Interestingly, none of these seven threonines aligned with the predicted phosphorylation motif for StkP substrates (EζDxζ[pT]xxΦK, in which ζ and Φ represent hydrophobic and hydrophilic amino acids) (35). This highlights the importance of conducting protein-specific studies to obtain a comprehensive phosphorylation pattern of a given bacterial protein. A similar conclusion can be drawn with regard to the StkP homologs PknB from *M. tuberculosis* and PrkC from *B. subtilis*, where their JMD may contain more phosphorylation sites than the 2 and 3 phosphorylation sites detected, respectively, by phosphoproteomics (36, 37). Given the absence of amino acid sequence similarity between bacterial JMDs, it can be inferred that the phosphorylation sites are also not conserved between the three homologs. This suggests that a JMD phosphorylation code, unique to each bacterial protein kinase, may be employed to regulate their function. Alternatively, several phosphorylation events could occur within the JMD, without strict location or number specificity, to provide the minimal negative charges that will influence the protein kinase activity.

In addition, we observed that the phosphorylation of the seven threonines in the JMD of StkP does not directly impact the kinase’s core function of substrate phosphorylation (Fig. 2B). It has been suggested that phosphorylation of the JMD in bacterial protein kinases may serve as a docking site for interacting with cellular partners containing specific recognition motifs (36, 37). A hallmark of phosphothreonine-specific recognition is the presence of proteins with ForkHead-Associated (FHA) domains (65). While not conserved in sequence, they share a similar 3D structure and are present across bacteria, archaea, and eukaryotes (66–68). FHA-containing proteins are notably found in several proteobacteria, including *Agrobacterium tumefaciens* and *Myxococcus xanthus,* as well as actinobacteria, such as *M. tuberculosis* and *Streptomyces coelicolor* (69, 70). Interestingly, an example of a specific interaction between a bacterial protein kinase and an FHA-containing protein is the interaction between the phosphorylated JMD of *M. tuberculosis* PknB and the FHA-containing protein FhaA (71). The latter participates in cell division and morphogenesis of *M. tuberculosis* (72). This observation supports the hypothesis that specific interactions between the phosphorylated JMD and cell division proteins may contribute to regulating cell division and morphogenesis of *S. pneumoniae*. However, surprisingly, FHA domains are not present in *streptococci,* and consequently in *S. pneumoniae*. This indicates that there are domains capable of interacting with the phosphorylated JMD that have yet to be characterized. Without being mutually exclusive, it is also possible that the interaction occurs in the absence of specific recognition motifs. These two hypotheses are supported by our proteomic analysis (Fig. 6), which demonstrates that the phosphomimetic version of the JMD facilitates the recruitment of proteins from the cell division machinery.

**Fig 7:**
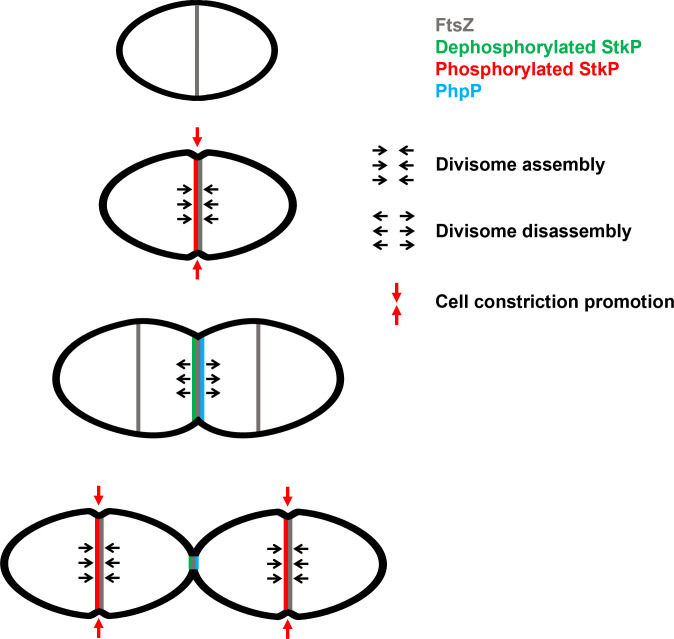
Model for the role of the phosphorylation of the JMD. In newborn cells, FtsZ (gray) is positioned at mid-cell and organizes the assembly of the divisome. Upon activation, StkP autophosphorylates at its seven threonine residues within the JMD (red), thereby facilitating the network of interactions between the division proteins to allow cell elongation and constriction (closed arrows). As the cell cycle progresses, dephosphorylation of StkP (green) by its cognate phosphatase PhpP (blue) favors the disassembly of the divisome (open arrows) and another cycle is ready to start again with the re-localization of FtsZ and the divisome at the division site of the daughter cells.

Our data revealed that JMD phosphorylation influences the timing of localization of StkP and FtsZ at the division septum (Fig. 3) and triggers early cell constriction (Fig. 4D). In addition, we found that phosphorylation of the JMD facilitates the recruitment of several cell division proteins (Fig. 6). While the precise molecular mechanism by which JMD phosphorylation influences these processes remains to be elucidated, our structural studies hint that phosphorylation does not induce a large conformational change within the JMD (Fig. 5). This leads us to speculate that phosphorylation may modify the dynamics of the interactions, potentially facilitating or inhibiting the interactions with other divisome components, and thereby regulating the assembly and disassembly of the division machinery. However, one intriguing observation is that the phosphorylation of the JMD of StkP, which is a late cell division protein, influences the timing of FtsZ localization, which is an early event at the division site. It was demonstrated that PhpP, the cognate phosphatase of StkP, is enriched at the division septum (38). We propose a model (Fig. 7) in which StkP JMD phosphorylation favors the interaction network within the divisome and, thus cell constriction. Upon dephosphorylation of StkP by PhpP, the interactions network is weakened, thereby resulting in the dissociation of the divisome during the final stages of the cell cycle and the subsequent relocalization at the cell equator, the future division site of the daughter cell. This model is consistent with our observations that JMD phosphorylation can compensate for the cell division block caused by inactive StkP, as phosphorylation of the JMD may counterbalance the absence of phosphorylation of StkP substrates (Fig. 4). Altogether, our findings show that the JMD may serve as a flexible regulatory scaffold for the assembly of the divisome.

The function of the JMD in kinase activation remains highly debated, even for eukaryotic kinase receptors (44). The field of bacterial protein kinases is still in its infancy, with much of our understanding coming from crystallographic studies of only a few kinases lacking their JMD (73–76). Although challenging, identifying the specific partners of the JMD and characterizing the structural organization of such complexes are now essential to further understand the contribution of StkP JMD phosphorylation to cell division and morphogenesis.

## MATERIALS AND METHODS

### Strains and growth conditions

*S. pneumoniae* R800, wild type (WT), and mutants were cultured in C + Y medium at pH 6.8 at 37°C. For growth on plates, THY-agar supplemented with 3% horse blood was used. Growth was monitored using a Tecan SUNRISE microtiter plate reader equipped with a 550 nm filter. Cells were initially grown to an OD_550_ of 0.1–0.2 in C + Y medium at pH 6.8. They were then diluted to an OD_550_ of 0.0001 and transferred to a Nunc Edge 96-well, non-treated, flat-bottom MicroplateGreen plate. Growth was monitored at 37°C with OD measurements taken every 10 min after brief shaking.

The *E. coli* DH5-α strain was used for cloning, and the *E. coli* BL21 Star (DE3) strain for protein overexpression. Both strains were cultured in Luria Bertani (LB) medium supplemented with appropriate antibiotics at 37°C, except for liquid-state NMR experiments, where the *E. coli* BL21 Star (DE3) strain was grown in M9 medium (77).

The strains and plasmids used in this study are listed in Table S3 and were verified by DNA sequencing.

### Allelic replacement mutagenesis and plasmid construction

To construct *S. pneumoniae* mutants, we employed a two-step procedure utilizing a bicistronic kan-rpsL cassette known as Janus, as previously detailed (78). All gene modifications were carried out at their native chromosomal locus in *S. pneumoniae*. DNA fragments of interest containing about 0.5–1 kb of chromosomal DNA flanking regions for homologous recombination were obtained by PCR using chromosomal DNA from *S. pneumoniae* with primers listed in Table S4. All transformations were performed with a separate reaction conducted without DNA as a negative control.

For plasmid construction, DNA fragments encoding the StkP phosphorylation-mutant constructs were generated by PCR, using chromosomal DNA from the *S. pneumoniae* R800 strain as a template. A detailed description of the primers used for the construction of strains and plasmids is provided in Table S4.

### Microscopy techniques and image analysis

For microscopy experiments, 0.5 µL of exponentially growing cells was placed onto a 1% agarose C + Y pad on a microscope slide and covered with a cover glass. The slides were then visualized using a Nikon Ti-E/B microscope equipped with an Orca-CMOS Flash4 V2 camera and a 100 Å ~1.45 objective. Images were acquired with NIS-Elements software (Nikon) and analyzed using ImageJ (http://rsb.info.nih.gov/ij/) and the MicrobeJ plugin (79). Statistical analysis was performed using Student’s *t*-tests, conducted in triplicate with the MicrobeJ plugin.

### Preparation of *S. pneumoniae* crude extracts and immunoblot analysis

Cultures of *S. pneumoniae* were grown in C + Y medium at pH 6.8 to an OD_550_, then pelleted, and resuspended in 10 mM Tris-HCl (pH 8), 1 mM EDTA, supplemented with a protease and phosphatase inhibitor cocktail (Sigma, P5726). After cell disruption by sonication, 20 µg of crude extracts was analyzed by SDS-PAGE and electro-transferred onto an Immobilon-P membrane (Millipore). Rabbit polyclonal primary antibodies were used at the following dilutions: 1:2,000 for anti-phosphothreonine (Cell Signaling Technology), 1:100,000 for anti-StkP-PASTA, 1:250,000 for anti-enolase in TBST-5% BSA, and 1:5,000 for anti-GFP (Amsbio) in TBST-1% BSA (15). The goat anti-rabbit HRP-conjugated secondary antibody (Bio-Rad) was used at a 1:5,000 dilution in TBST-BSA 1%.

### Protein production and purification

The pETPhos vectors used encode the catalytic domain of StkP from residues 1 to 287 (StkP_KD_), the phosphorylation mutants of the cytoplasmic domain of StkP from residues 1 to 344 (StkP_cyto_-7TA and StkP_cyto_-7TE), and the phosphorylation mutants of the juxtamembrane domain of StkP from residues 270 to 344 (JMD-wt, JMD-7TA and JMD-7TE). The recombinant plasmids were transformed into the *E. coli* BL21 Star (DE3) strain. The strains were grown in LB medium for SAXS experiments and in M9 medium supplemented with ^15^NH_4_Cl or ^13^C-labeled glucose and ^15^NH_4_Cl for NMR experiments at 37°C until reaching an OD_600_ of 0.6 (77). Protein production was induced with 1 mM isopropyl-β-D-thiogalactopyranoside (IPTG) for 3 h at 37°C. Bacterial pellets were then resuspended in buffer A (25 mM Tris-HCl, pH 8, 300 mM NaCl, 10% [vol/vol] glycerol, 10 mM imidazole) supplemented with 1 µg/mL lysozyme, 6 µg/mL DNase/RNase, and a 1× Roche protease inhibitor. After sonication and centrifugation at 15,000 × *g* for 30 min, the supernatant was loaded onto a Ni-NTA agarose resin (Qiagen). Fractions containing the overexpressed protein were pooled and dialyzed overnight at 4°C against a buffer containing 25 mM Tris (pH 8), 300 mM NaCl, 1 mM DTT, and 0.5 mM EDTA, after adding a solubility-enhanced hexahistidine-tagged L56V/S135G tobacco etch virus (TEV) protease in a 20:1 ratio (80). The uncleaved protein and TEV protease were separated from the cleaved protein by a second purification step on a Ni-NTA agarose resin. The flow-through containing the cleaved protein was concentrated and loaded onto a HiLoad 16/600 Superdex 200 column (Cytiva) equilibrated in a buffer containing 25 mM Bis-Tris (pH 7.4) and 150 mM NaCl for size exclusion chromatography purification. Fractions containing the pure protein were pooled and concentrated. Protein purity was assessed by SDS-PAGE, and the monodispersity of the samples was verified by dynamic light scattering (DLS) using a Zetasizer Nano S (Malvern Instruments) after centrifugation at 16,000 × *g* for 10 min at 4°C. For long-term storage, samples were rapidly frozen in liquid nitrogen and stored at −80°C.

### NMR resonance assignments of JMD peptides

The 2D-NMR experiments were collected on 300 µM of ^15^N-labeled StkP-JMD-wt, StkP-JMD-7TA, and StkP-JMD-7TE samples while 3D-NMR experiments were conducted on 300 µM of ^13^C,^15^N-labeled StkP-JMD-7TA and StkP-JMD-7TE samples. All NMR samples were prepared in 25 mM Bis-Tris, 150 mM NaCl buffer at pH 7.4 and containing 10% D_2_O. Backbone resonance assignments were performed using a combination of 2D ^1^H-^15^N-BEST-TROSY and 3D HNCA, HNCO, HNCACO, HNCACB, iHNCACB, HNCOCANH, and HNCACONH experiments for StkP-JMD-7TA, while HNCOCAB, HNCACO, HNCACB, HNCANH, HNCOCANH, and HNCO experiments were used for StkP-JMD-7TE. To minimize the number of overlaps, the BEST-TROSY version of the above-listed experiments was used (81, 82). All spectra were recorded at 5°C using Bruker AVANCE spectrometers operating at 600, 700, and 850 MHz proton frequencies equipped with TCI cryoprobes. NMR spectra were processed using the TopSpin software by Bruker in its 3.2 version and were analyzed using the CcpNmr Analysis software (83). The SSP (Secondary Structure Propensity) program was used to estimate the residue-specific secondary structure propensities of StkP-JMD-7TA and StkP-JMD-7TE based on their assigned ^13^C (C_α_, C_β_) chemical shifts (47).

### Small-angle X-ray scattering (SAXS) data collection, processing, and modeling

SAXS data were collected on beamline BM29 from the European Synchrotron Radiation Facility in Grenoble (France). Samples at a concentration of 2 mg/mL were centrifuged at 14,000 × *g* for 10 min before being injected into the BM29 flow cell. Scattering data were collected at 4°C, with 10 frames recorded at an exposure time of 1 second per frame (84). Buffer background scattering data were also collected using the gel filtration buffer from the sample purification process. Data processing, including background subtraction, averaging, and scaling, was performed using the EDNA pipeline available at the beamline. ScÅtter IV (85) was utilized to generate the pair distribution function P(r) and to determine Dmax and Rg from the scattering curves I(q) versus q, in an automated, unbiased manner.

The analysis of StkP_cyto_-7TA, based on the ensemble optimization Method, was performed using the EOM program (86). We used a model of the kinase domain of StkP (residues 1–270) obtained from AlphaFold3 (87). Initially, 10,000 protein structures were generated for each protein construct, with the intrinsically disordered region (IDR) modeled as a random chain within Ramachandran constraints. Scattering profiles were calculated for each model in the 10,000-member ensembles. A genetic algorithm was then used to select sub-ensembles that best described the SAXS data. This involved fitting the SAXS data with a combinatorial, volume-fraction-weighted sum of individual model scattering profiles from the initial pool of structures.

### Immunoprecipitation and mass spectrometry analysis

GFP-StkP or GFP-StkP-7TA and GFP-StkP-7TE were immunoprecipitated to identify the phosphorylation sites within the JMD or the protein partners, respectively. The *gfp-stkP* strain was grown at 37°C in THY medium in the absence or presence of ampicillin (1 µg/mL for 15 min) until reaching an OD_550_ of 0.4. *gfp-stkP-7TA* and *gfp-stkP-7TE* strains were grown at 37°C in C + Y medium until reaching an OD_550_ of 0.4. The cultures were then centrifuged at 5,000 × *g* for 15 min at 4°C. The resulting cell pellets were incubated at 30°C for 15 min in buffer A (0.1 M Tris-HCl, pH 7.5, 2 mM MgCl_2_, 1 M sucrose, 6 mg/mL DNase I and RNase A, and 1 µg/mL protease inhibitor). This step was repeated twice before a final centrifugation. The pellet was then resuspended and incubated for 30 min at room temperature in buffer A supplemented with 800 U of mutanolysin and 8 mg/mL of lysozyme. Following another centrifugation at 5,000 × *g* for 10 min at 4°C, the pellet was resuspended in 2 mL of cold buffer B (0.1 M Tris-HCl, pH 7.5, 1 mM EDTA, 1% digitonin, 6 mg/mL DNase I and RNase A, and 1 µg/mL protease inhibitor) and incubated for 30 min at 37°C. The lysate was then incubated for 2 h at 4°C with the GFP-trap slurry, as recommended by the GFP-Trap A Chromotek protocol. After centrifugation at 2,700 × *g* for 2 min at 4°C, the pellet was washed three times in a buffer containing 10 mM Tris-HCl, 150 mM NaCl, 0.5 mM EDTA, and 0.05% Digitonin, and five times in a 50 mM ammonium bicarbonate buffer. To quantify the protein partners of GFP-StkP-7TA compared to GFP-StkP-7TE, the sample was directly analyzed by mass spectrometry. To determine the phosphorylated threonine residues in the JMD, the sample was analyzed by SDS PAGE, and GFP-StkP-7TA or GFP-StkP-7TE were cut out from the gel and analyzed by mass spectrometry. A comprehensive description of sample preparation and mass spectrometry protocols is available in the supplemental materials.

## Data Availability

The mass spectrometry phosphoproteomics data have been deposited to the ProteomeXchange Consortium via the PRIDE (88) partner repository with the data set identifier PXD052685. The mass spectrometry proteomics data have been deposited to the Center for Computational Mass Spectrometry repository (University of California, San Diego) via the MassIVE tool with the data set identifier MassIVE MSV000096495.

## SUPPLEMENTAL MATERIAL

Supplemental material mbio.03799-24-s0001.pdf Figures S1 to S7; Tables S1, S2, and S4; supplemental materials and methods.

Table S2 mbio.03799-24-s0002.xlsx Proteomic analysis of GFP-StkP-7TA and GFP-StkP-7TE partners.

ASM does not own the copyrights to Supplemental Material that may be linked to, or accessed through, an article. The authors have granted ASM a non-exclusive, world-wide license to publish the Supplemental Material files. Please contact the corresponding author directly for reuse.
