# Supplementary material for: The juxtamembrane domain of StkP is phosphorylated and influences cell division in Streptococcus pneumoniae
Source: mBio. 2025 Apr 8;16(5):e03799-24. doi: 10.1128/mbio.03799-24 (PMC12077195; doi:10.1128/mbio.03799-24)
Supplement: Supplemental material — Figures S1 to S7; Tables S1, S2, and S4; supplemental materials and methods. [file mbio.03799-24-s0001.pdf]

SUPPLEMENTAL MATERIAL

The juxtamembrane domain of StkP is phosphorylated and influences cell division in *Streptococcus pneumoniae*

Mélisse Hamidi[1], Sathya Narayanan Nagarajan[1, #], Vaishnavi Ravikumar[2], Virginie Gueguen-Chaignon[3], Cédric Laguri[4], Céline Freton[1], Ivan Mijakovic[2], Jean-Pierre Simorre[4], Stéphanie Ravaud[1,†], Christophe Grangeasse[1, †,*]

[1] Molecular Microbiology and Structural Biochemistry, UMR 5086, Université Claude Bernard Lyon 1, CNRS, Lyon, France

[2] Chalmers University of Technology, Department of Biology and Biological Engineering, Gothenburg, Sweden

[3] Protein Science Facility, CNRS UAR3444, INSERM US8, Université Claude Bernard Lyon 1, Ecole Normale Supérieur de Lyon, Lyon, France

[4] Institut de Biologie Structurale, CEA, CNRS UMR 5075, Université Grenoble Alpes, 3800, Grenoble, France

Figure S1 to S7

Table S1, S3 and S4

Supplemental Materials and Methods for proteomics and phosphoproteomics

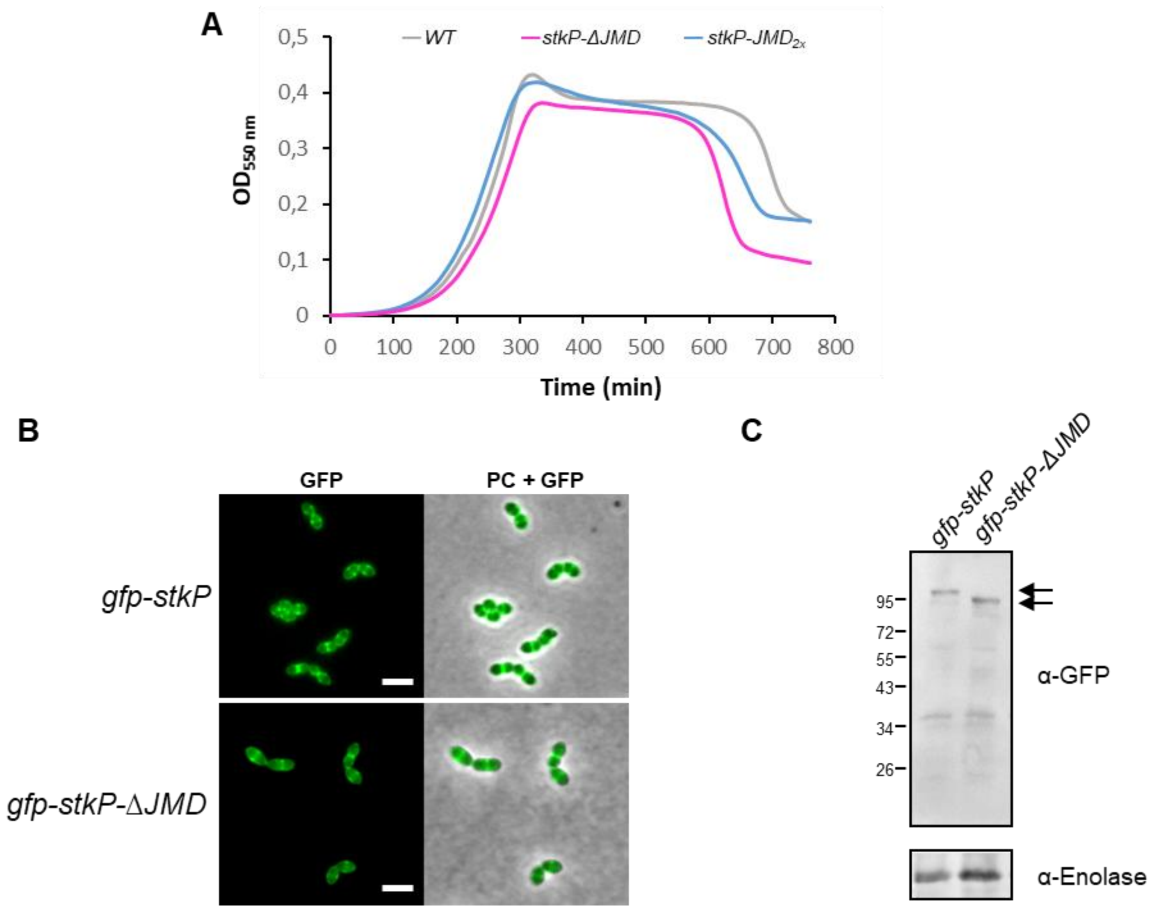

Figure S1: Analysis of *WT*, *stkP-ΔJMD*, *stkP-JMD*$_{2x}$, *gfp-stkP*, *gfp-stkP-JMD*$_{2x}$ strains. (A) Growth of *WT*, *stkP-ΔJMD* and *stkP-JMD*$_{2x}$ strains in C + Y medium. Experiments were performed in triplicate. **(B)** Localization of GFP–StkP and GFP-StkPΔJMD. GFP fluorescent signal (left row) and overlay between phase-contrast and GFP images (right row) are shown. Scale bar, 2 µm. **(C)** Expression of *gfp-stkP* and *gfp-stkP-ΔJMD*. The Western immunoblot was probed with anti-GFP antibodies (α-GFP) (upper panel). Detection with anti-enolase antibodies (α-Enolase) (lower panel) was carried out as a loading control.

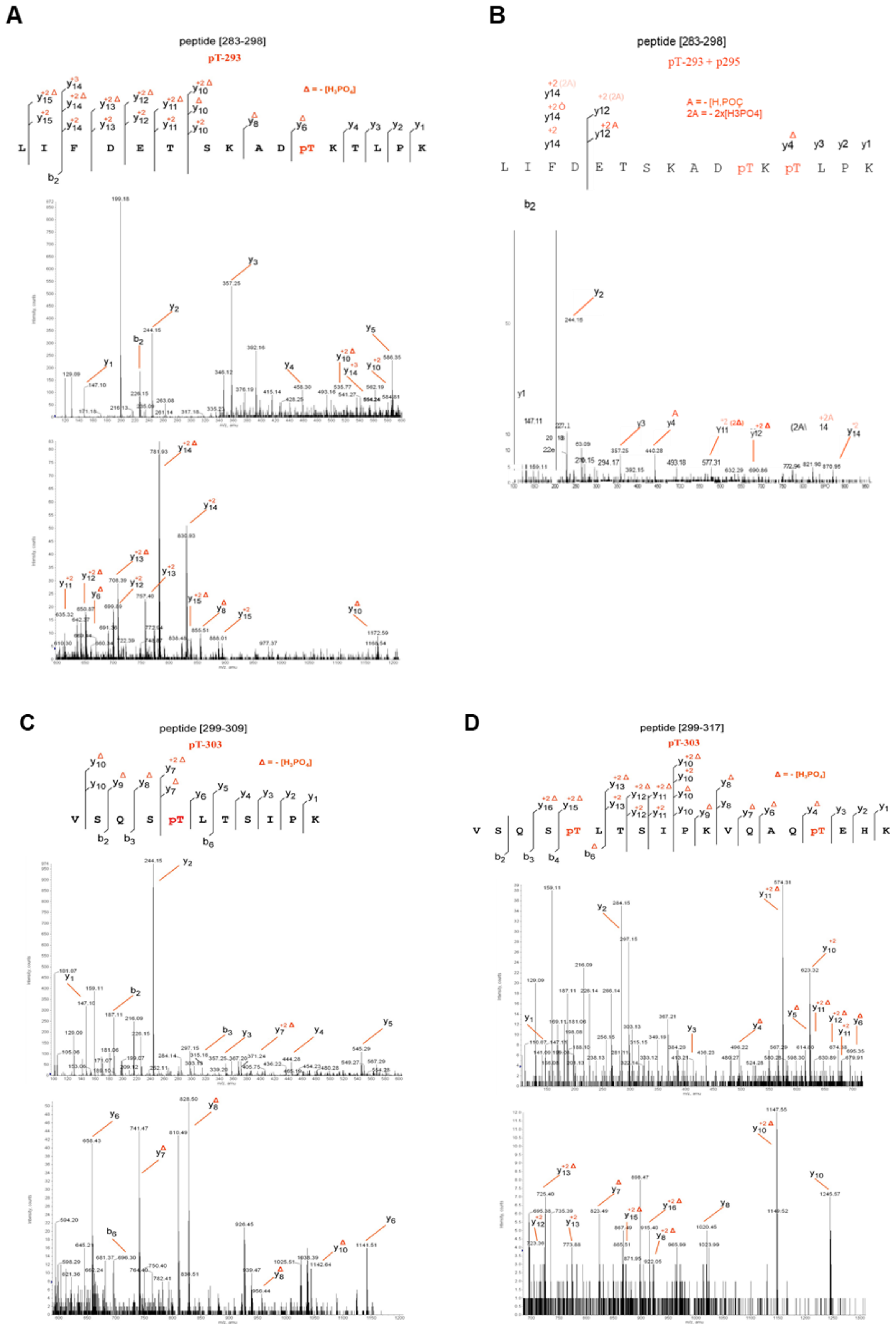
A
peptide [283-298]
pT-293
Δ = - [H3PO4]
L I F D E T S K A D pT K T L P K
B
peptide [283-298]
pT-293 + p295
L I F D E T S K A D pT K pT L P K
C
peptide [299-309]
pT-303
Δ = - [H3PO4]
V S Q S pT L T S I P K
D
peptide [299-317]
pT-303
Δ = - [H3PO4]
V S Q S pT L T S I P K V Q A Q pT E H K

E

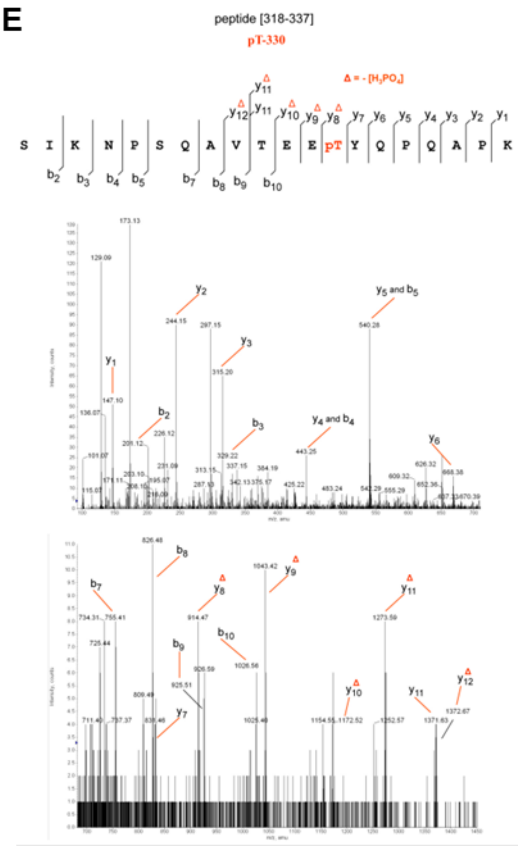

Figure S2: Identification of the JMD phosphothreonines in cells grown in absence of ampicillin. (A) Thr-293 phosphorylation: MS/MS mass spectrum of the charged ions of phosphopeptide 283-298 at **m/z** 629.63 (monoisotopic mass: 1889.99 Da). **(B)** Thr-293 and Thr-295 phosphorylation: MS/MS mass spectrum of the charged ions of phosphopeptide 283-298 at **m/z** 656.29 (monoisotopic mass: 1965.92 Da). **(C)** Thr-303 phosphorylation: MS/MS mass spectrum of the charged ions of phosphopeptide 299-309 at **m/z** 620.773 (monoisotopic mass: 1239.62 Da). **(D)** Thr-303 and Thr-314 phosphorylation: MS/MS mass spectrum of the charged ions of phosphopeptide 299-317 at **m/z** 748.04 (monoisotopic mass: 2241.11 Da). **(E)** Thr-330 phosphorylation: MS/MS mass spectrum of the charged ions of phosphopeptide 318-337 at **m/z** 766.05 (monoisotopic mass: 2195.14 Da).

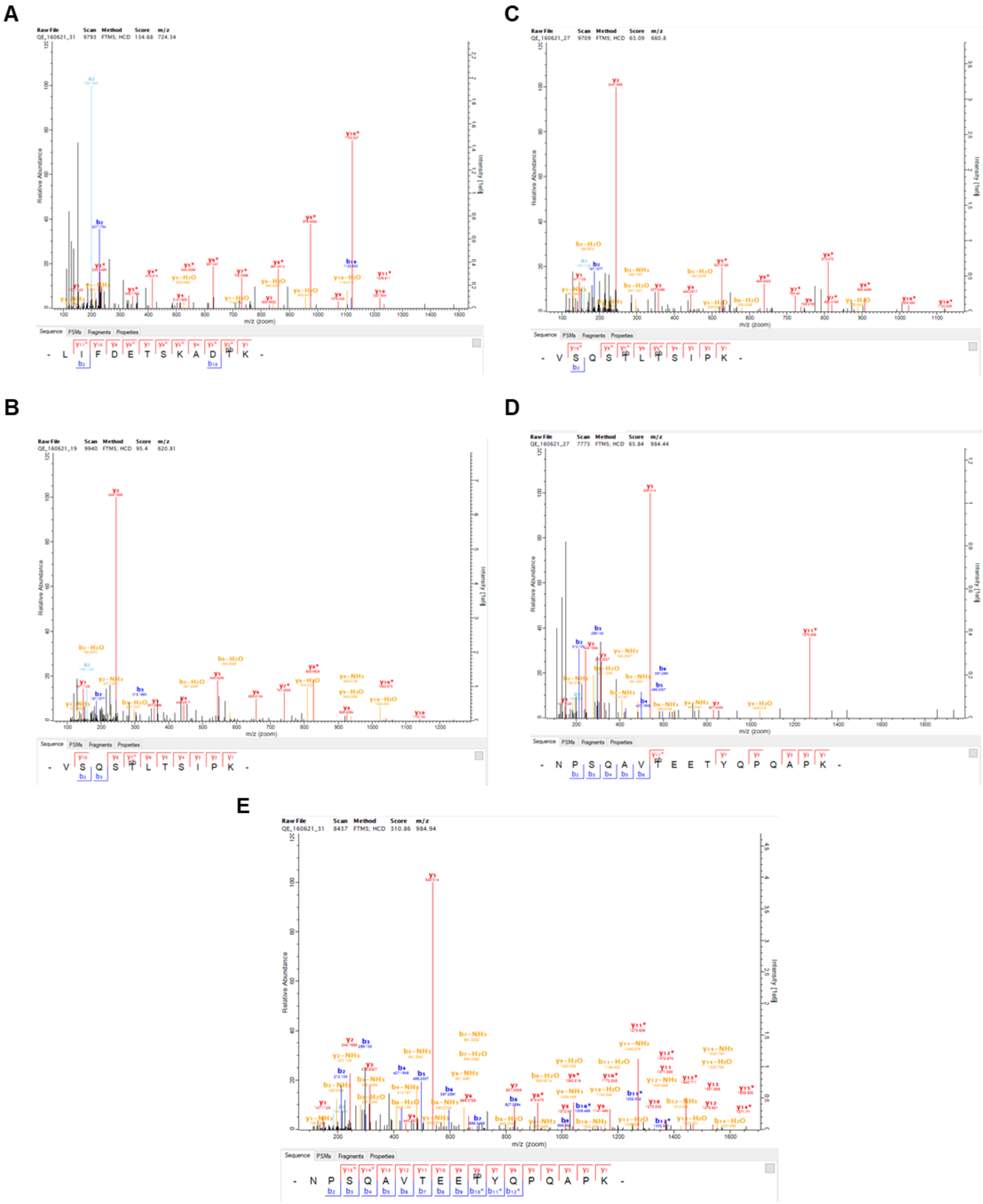
A
B
C
D
E
Raw File
Scan
Method
Score
m/z
Relative Abundance
Intensity [1e6]
m/z (zoom)
Sequence
PSMs
Fragments
Properties
- L I F D E T S K A D T K -
- V S Q S T L T S I P K -
- N P S Q A V T E E T Y Q P Q A P K -

Figure S3: Identification of the JMD phosphothreonines in cells grown in presence of ampicillin. **(A)** Thr-293 phosphorylation: MS/MS mass spectrum of the charged ions of phosphopeptide 283-294 at **m/z** 724.34. **(B)** Thr-303 phosphorylation: MS/MS mass spectrum of the charged ions of phosphopeptide 299-309 at **m/z** 620.81. **(C)** Thr-305 phosphorylation: MS/MS mass spectrum of the charged ions of phosphopeptide 299-309 at **m/z** 660.8. **(D)** Thr-327 phosphorylation: MS/MS mass spectrum of the charged ions of phosphopeptide 321-337 at **m/z** 984.44. **(E)** Thr-330 phosphorylation: MS/MS mass spectrum of the charged ions of phosphopeptide 321-337 at **m/z** 984.94.

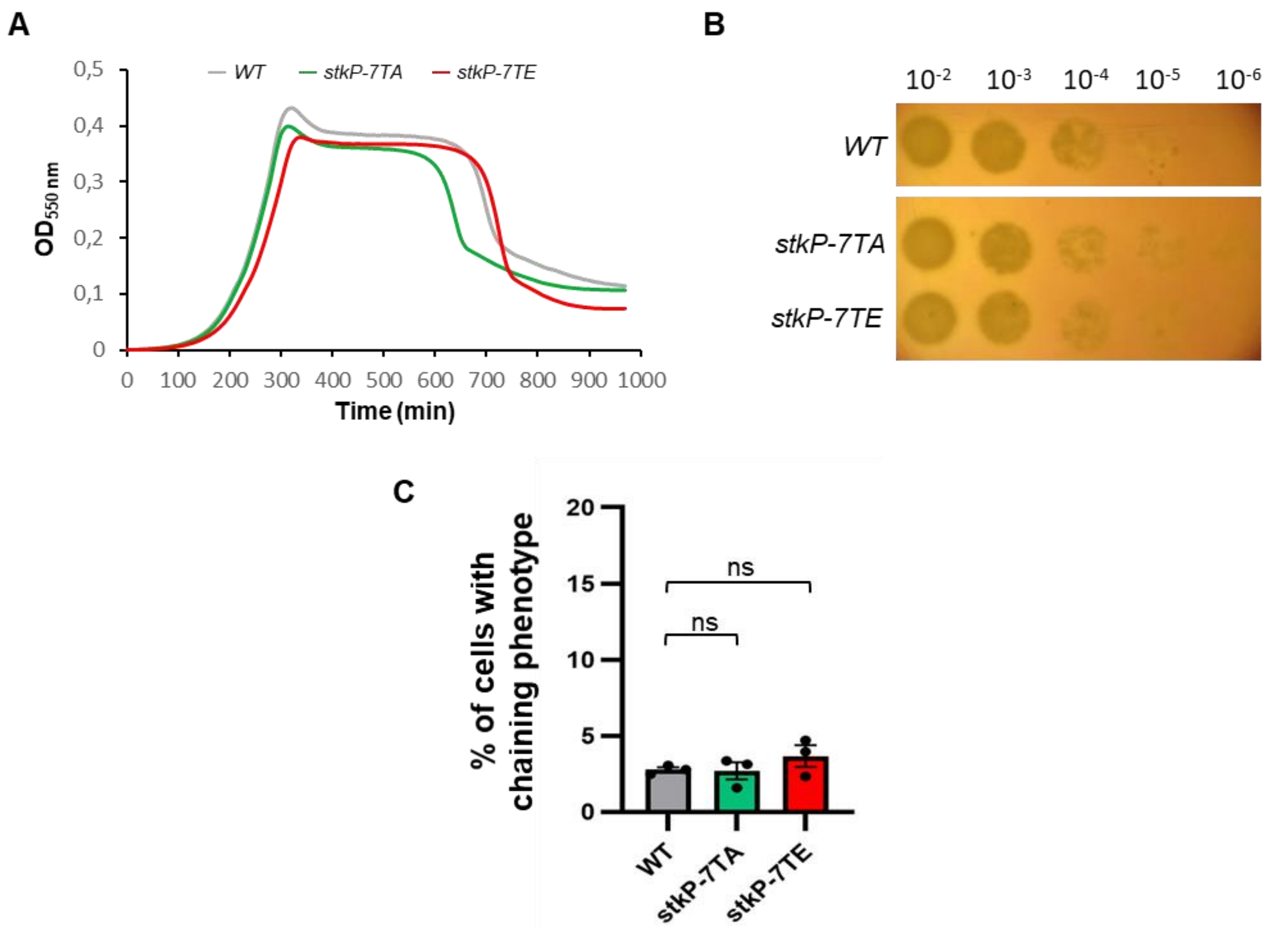

Figure S4: Characterization of *WT*, *stkP-7TA* and *stkP-7TE* strains. (A) Growth of *WT*, *stkP-7TA* and *stkP-7TE* strains in C + Y medium. Experiments were performed in triplicate. **(B)** Cell viability assays. *WT*, *stkP-7TA* and *stkP-7TE* strains were grown to exponential phase and normalized to an OD_{550} of 0,1. The cultures were serially diluted, and 10 µl of each dilution was spotted onto THY horse blood plates. Images shown are representative of three independent experiments. **(C)** Bar chart representing the percentage of pneumococcal cells harboring a chaining phenotype (minimum five cells per chain). Bars represent the mean (± SEM) of three independent experiments. The WT was compared separately with the *stkP-7TA* or the *stkP-7TE* mutants with an unpaired t-test (ns P > 0,05).

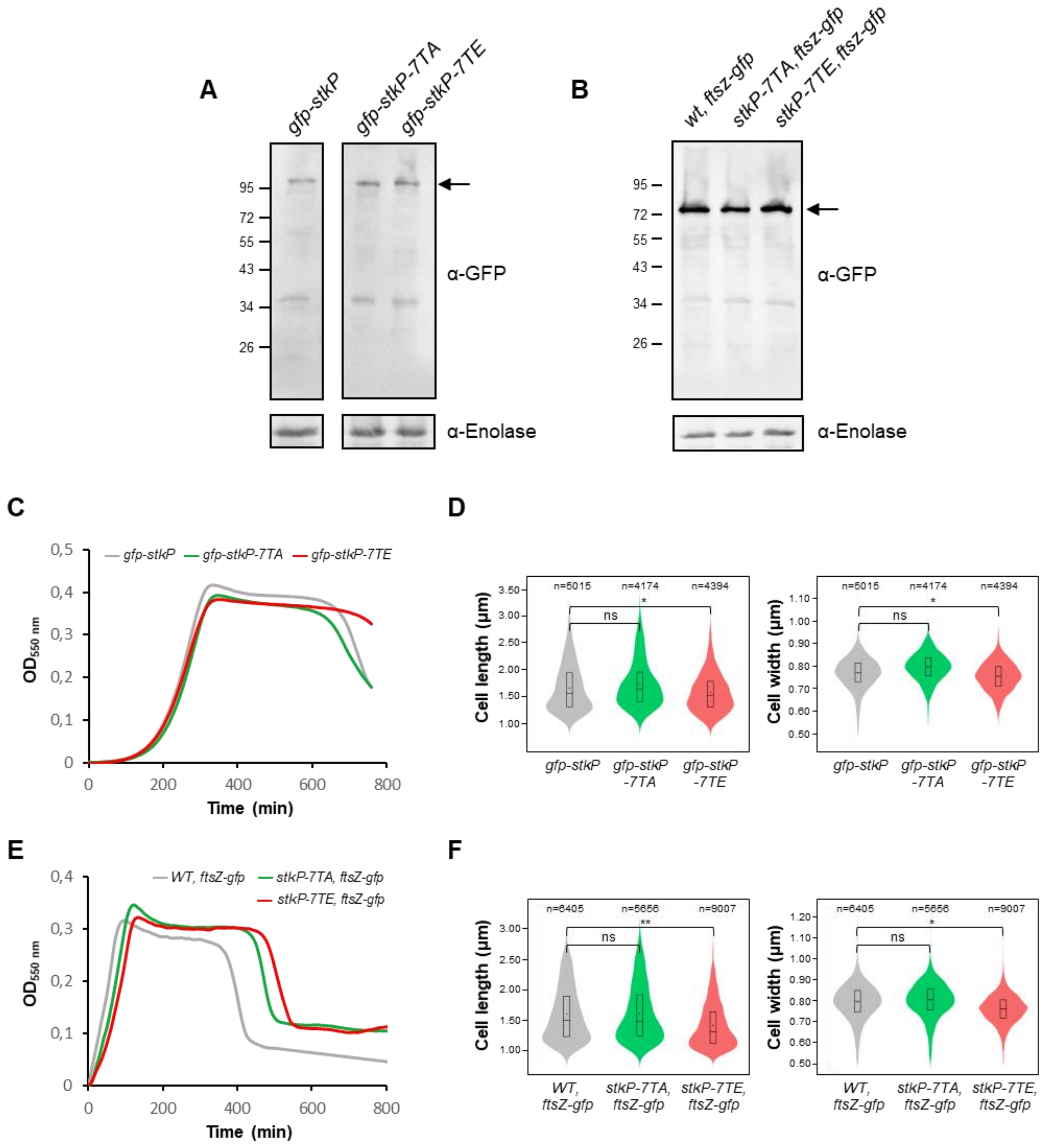

Figure S5: Analysis of strains coding for *stkP* derivatives and/or fusions and *ftsZ-gfp*. (A) Expression of *gfp-stkP*, *gfp-stkP-7TA* and *gfp-stkP-7TE*. The Western immunoblot was probed with anti-GFP antibodies (α-GFP) (upper panel) and anti-enolase antibodies (α-Enolase) (lower panel) used as a loading control. **(B)** Expression of *ftsZ-gfp* in *WT*, *ftsZ-gfp*; *stkP-7TA*, *ftsZ-gfp*; and *stkP-7TE, ftsZ-gfp* cells. The Western blot was probed with anti-GFP antibodies (α-GFP)

(upper panel) and anti-enolase antibodies (α-Enolase) (lower panel). **(C)** Growth of *gfp-stkP*, *gfp-stkP-7TA* and *gfp-stkP-7TE* strains in C + Y medium Experiments were performed in triplicate. **(D)** Violin plot showing the distribution of the cell length (left panel) and cell width (right panel) for *gfp-stkP* (grey), *gfp-stkP-7TA* (green) and *gfp-stkP-7TE* (red) strains as determined using MicrobeJ. The box indicates the 25th to the 75th percentile. The mean and the median are respectively indicated with a dot and a line in the box. Statistical comparison was done using t-test (ns P > 0,05 and *P < 0.05). The n values represent the number of cells analyzed. Experiments were performed in four replicates. **(E)** Growth of *ftsZ-gfp* in *WT, ftsZ-gfp*; *stkP-7TA, ftsZ-gfp*; and *stkP-7TE, ftsZ-gfp* strains in C + Y medium. Experiments were performed in triplicate. **(F)** Violin plot showing the distribution of the cell length (left panel) and cell width (right panel) for *WT, ftsZ-gfp* (grey), *stkP-7TA, ftsZ-gfp* (green) and *stkP-7TE, ftsZ-gfp* (red) strains as determined using MicrobeJ. The box indicates the 25th to the 75th percentile. The mean and the median are respectively indicated with a dot and a line in the box. Statistical comparison was done using t-test (ns P > 0,05, *P < 0.05 and **P < 0.01). The n values represent the number of cells analyzed. Experiments were performed in four replicates.

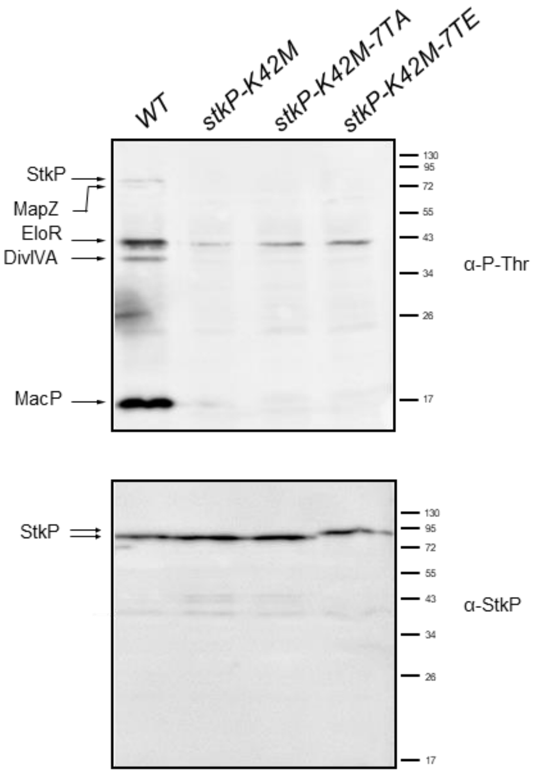

Figure S6: Phosphorylation patterns of *WT*, *stkP-K42M*, *stkP-K42M-7TA* and *stkP-K42M-7TE* strains. Western immunoblots of whole-cell lysates from *WT*, *stkP-K42M*, *stkP-K42M-7TA* and *stkP-K42M-7TE* cells were probed with antiphosphothreonine antibodies (upper panel). Black arrows indicate StkP and its substrates MapZ, DivIVA, EloR and MacP. In the lower panel, western immunoblot of whole-cell lysates were probed with anti-StkP PASTA. Images are representative of experiments made in triplicates.

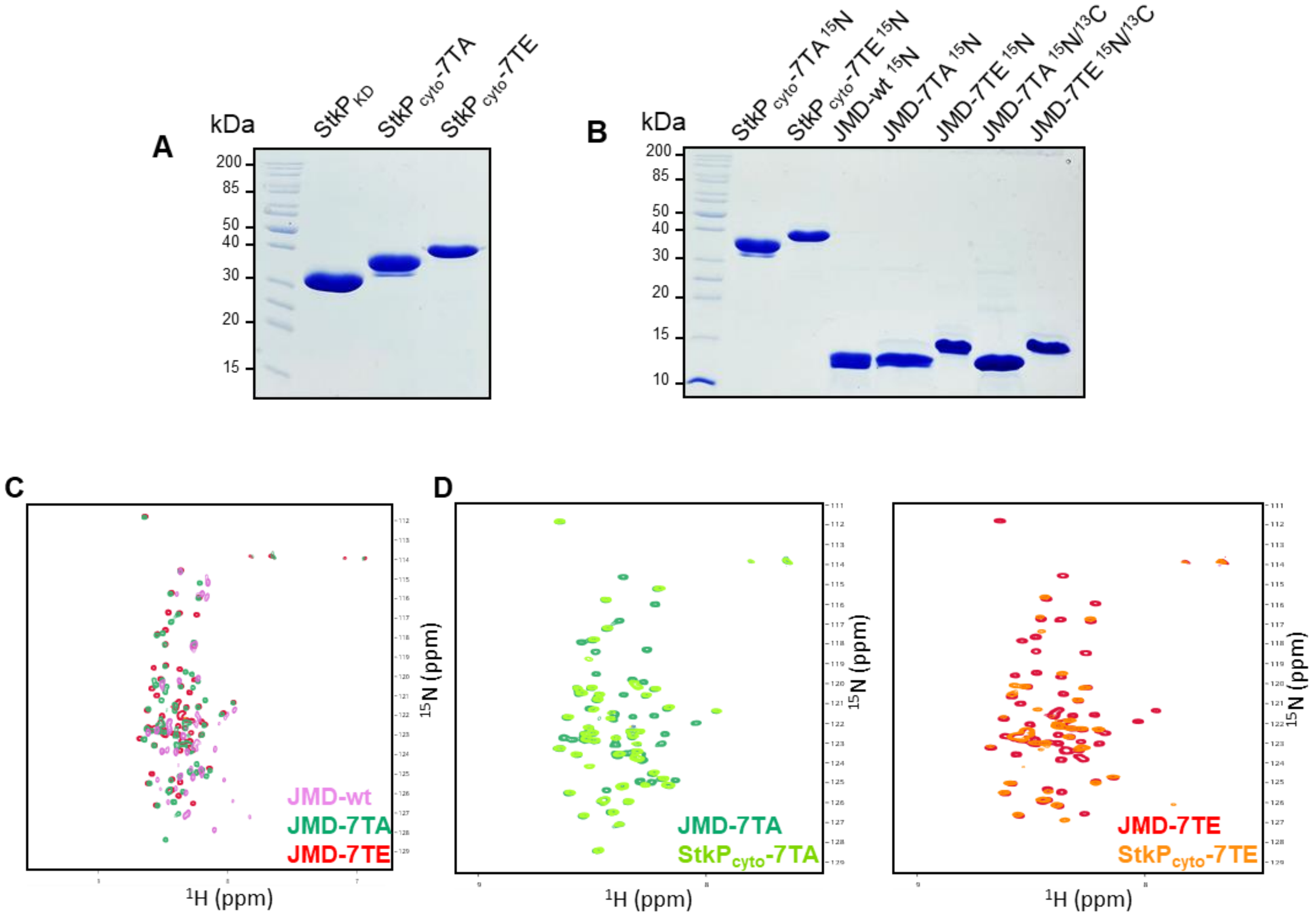

Figure S7: Analysis of StkP derivatives by SAXS and NMR. (A) Purification and analysis by SDS-PAGE of StkP derivatives used in the SAXS study. **(B)** Same as (A) for StkP derivatives used in the NMR study. **(C)** Overlay of the ^{1}H–^{15}N BEST-TROSY spectra recorded for ^{15}N-labeled JMD-WT (magenta), JMD-7TA (green) and JMD-7TE (red). A very low chemical shift dispersion is observed between these spectra. **(D)** Overlay of the ^{1}H–^{15}N BEST-TROSY spectra recorded for ^{15}N-labeled JMD-7TA (green) and StkP$_{cyto}$-7TA (light green) (left panel) and for ^{15}N-labeled JMD-7TE (red) and StkP$_{cyto}$-7TE (orange) (right panel). The high degree of peak alignment between StkP$_{cyto}$ and the JMD in both overlays indicates structural similarity.

Table S1: Small angle X-ray scattering data collection, processing and data analysis of $StkP_{KD}$ $StkP_{cyto}$-7TA and $StkP_{cyto}$-7TE.

	**$StkP_{KD}$**	**$StkP_{cyto}$-7TA**	**$StkP_{cyto}$-7TE**
**Data collection parameters:**			
Q Range ($Å^{-1}$)	0.0162 - 0.2365	0.0120 - 0.1746	0.01 - 0.1849
**Structural parameters:**			
I(0) (a.u.) (from Guinier)	101.1 ± 0.21	58.98 ± 0.24	72.01 ± 0.28
Rg (Å) (from Guinier)	26.04 ± 0.26	31.66 ± 0.95	30.86 ± 0.58
I(0) (a.u.) (from P(r))	99.21	48.34	64.03
Rg (Å) (from P(r))	25.98	29.55	30.77
Dmax (Å)	87.0	113.0	111.5
Porod volume estimation ($Å^3$)	106.086	156.426	176.255

Table S3: Strains and plasmids used in this study

Strain	Genotype and description	Reference	Primers (Table S2)
S. pneumoniae strains			
R800	*R800 rpsL1; Str^R*	Gift from J.-P. Claverys (France)	
Wild type	*R800 rpsL1; Str^R*	(1)	
StkP::kan-rpsL	*R800 rpsL1, StkP::kan-rpsL; Kan^R*	(1)	
GFP-StkP	*R800 rpsL1, GFP-StkP; Str^R*	(1)	
StkPΔJMD	*R800 rpsL1, StkPΔJMD(R277-P333); Str^R*	This study	3, 4, 5 and 6
$StkPJMD_{2x}$	*R800 rpsL1, $StkPΔJMD_{2x}$ (S271-M243); Str^R*	This study	3, 4, 27 and 28
GFP-StkPΔJMD	*R800 rpsL1, GFP-StkPΔJMD; Str^R*	This study	3, 4, 7 and 8
StkP-7TA	*R800 rpsL1, StkP-7TA (T293A, T295A, T303A, T305A, T314A, T327A, T330A); Str^R*	This study	12, 13, 9,10 and 11
GFP-StkP-7TA	*R800 rpsL1, GFP-StkP-7TA; Str^R*	This study	12, 13, 7 and 8
StkP-7TE	*R800 rpsL1, StkP-7TE (T293E, T295E, T303E, T305E, T314E, T327E, T330E); Str^R*	This study	3, 4, 15, 16 and 17
GFP-StkP-7TE	*R800 rpsL1, GFP-StkP-7TE; Str^R*	This study	3, 4, 7 and 14
FtsZ-GFP	*R800 rpsL1, FtsZ-GFP; Str^R*	(2)	
StkP-7TA, FtsZ-kan-rpsl	*R800 rpsL1, StkP-7TA, FtsZ-kan-rpsL; Str^R*	This study	1,2,18 and 19
StkP-7TA, FtsZ-GFP	*R800 rpsL1, StkP-7TA, FtsZ-GFP; Str^R*	This study	18 and 19
StkP-7TE, FtsZ-kan-rpsl	*R800 rpsL1, StkP-7TE, FtsZ-kan-rpsL; Str^R*	This study	1,2,18 and 19
StkP-7TE, FtsZ-GFP	*R800 rpsL1, StkP-7TE, FtsZ-GFP; Str^R*	This study	18 and 19
StkP-K42M	*R800 rpsL1, StkP-K42M; Str^R*	(2)	
StkP-K42M-7TA	*R800 rpsL1, StkP-K42M-7TA (T293A, T295A, T303A, T305A, T314A, T327A, T330A); Str^R*	This study	12, 13, 8 and 14
StkP-K42M-7TE	*R800 rpsL1, StkP-K42M-7TE (T293E, T295E, T303E, T305E, T314E, T327E, T330E); Str^R*	This study	12, 13, 8 and 14
E. coli strains			
DH5α	*F– φ80lacZΔ M15 Δ (lacZYA-argF) U169 recA1 endA1 hsdR17 (r_K– m_K+) PhoA supE44 λ- thi–1 gyrA96 relA1*	Lab collection	
BL21 star (DE3)	F – ompT gal dcm lon $hsdS_B$(r_B –m_B –) λ(DE3 [lacI lacUV5-T7p07 ind1 sam7 nin5]) $[malB^+]_{K-12}$($λ^S$)	Invitrogen	
Plasmids			
$pETPhos\text{-}StkP_{kinase}$	*pETPhos derivative, encoding StkP, from Met1 to Glu287, Amp^R*	This study	21, 22, 23 and 24
pETPhos-StkP-7TA	*pETPhos derivative, encoding StkP-7TA (T293A, T295A, T303A, T305A, T314A, T327A, T330A), from Met1 to Arg344, Amp^R*	This study	21, 23, 24 and 25

pETPhos-StkP-7TE	*pETPhos derivative, encoding StkP-7TE (T293E, T295E, T303E, T305E, T314E, T327E, T330E), from Met1 to Arg344, Amp^R*	This study	21, 23, 24 and 25
pETPhos-StkP-JMD-wt	*pETPhos derivative, encoding StkP-JMD-wt, from Ser270 to Arg344, Amp^R*	This study	23, 24, 26 and 25
pETPhos-StkP-JMD-7TA	*pETPhos derivative, encoding StkP-JMD-7TA (T293A, T295A, T303A, T305A, T314A, T327A, T330A), from Ser270 to Arg344, Amp^R*	This study	23, 24, 26 and 25
pETPhos-StkP-JMD-7TE	*pETPhos derivative, encoding StkP-JMD-7TE (T293E, T295E, T303E, T305E, T314E, T327E, T330E), from Ser270 to Arg344, Amp^R*	This study	23, 24, 26 and 25

Table S4: Primers used in this study

#	Primer Name	+/-	Sequence 5'→3'
1	5'- [kan-rpsL]	+	CCGTTTGATTTTTAATGGATAATG
2	3' - [kan-rpsL]	-	AGAGACCTGGGCCCCTTTCC
3	upstream region StkP	+	GGTCAGGTTACTGCTCTGGAC
4	downstream region StkP	-	GAAAGGAGCACTAAAGGTCGC
5	*5'-StkPΔJMD*	+	CAAGCACCGAAAAAACATAG
6	*3'-StkPΔJMD*	-	CTTAAATCTATGTTTTTTCGGTGCTTGATTGTAGGACAAGCTACTAGAC
7	linker GFP-StkP	+	CTCGAGGGATCCGGAATGATCCAAATCGGCAAGATTTTTG
8	downstream GFP-StkP	-	CATGGCATAGATATCACTCTGC
9	StkP-5TA	+	GGTTTCTCAGAGTgCCTTGgCATCTATTCCTAAGGTTCAAGCGCAGgCAGAACACAAATCAATCAAAAACCCAAGCCAGGCTGTGgCAGAGGAAgCTTACCAACCACAAGC
10	StkP-2TA	-	CGGCAAGGCCTTGGCATCTGC
11	StkP-4TA	+	GATgCCAAGgCCTTGCCGAAGGTTTCTCAGAGTgCCTTGgCATCTATTCCTAAGGTTCAAGCGCAG
12	upstream region StkP	+	GTTGATACCCAGATCGATACAG
13	downstream region StkP	-	AGGCAAAACCAAATAAGGTCG
14	downstream GFP-StkP	+	GGATTGCTGTAGCCTTTGCAGAGAC
15	StkP-T330E	+	GGAAGAATACCAACCACAAGCACCG
16	StkP-M1	+	ATGATCCAAATCGGCAAGATTTTTG
17	StkP-R344	-	ACGCATCTTAAATCTATGTTTTTTCGGTGC
18	upstream region FtsZ	+	CCTATCCGCCTCTTGCAAGC
19	downstream region FtsZ	-	CTTTTAAAGACATGGTTCTCTCCTAC
20	StkP-K42M	-	CCTCAGAACCATCACTGCCAC
21	StkP- pETPhos	+	GTATTTCCAGGGCCATATGATCCAAATCGGCAAGATTTTTG
22	StkP-E287-pETPhos	-	CTTAGTTATTAGGATCCTTCATCAAAGATTAACTTACTTTC
23	pETPhos	-	CATATGGCCCTGGAAATACAAG
24	pETPhos	+	GGATCCTAATAACTAAGTAAACTAGTGC
25	StkP-R344-pETPhos	-	CTTAGTTATTAGGATCCACGCATCTTAAATCTATGTTTTTTCGGTGC
26	StkP-S271-pETPhos	+	GTATTTCCAGGGCCATATGAGTAGCTTGTCCTACAATCGTAG
27	*5'-StkPJMD$_{2x}$*	+	CATAGATTTAAGATGAGTAGCTTGTCCTACAATCG
28	*3'-StkPJMD$_{2x}$*	-	CGATTGTAGGACAAGCTACTCATCTTAAATCTATG

SUPPLEMENTAL MATERIALS AND METHODS

To determine the phosphorylated threonine in the JMD, the gel pieces were first destained in 50 % acetonitrile/5 mM ammonium bicarbonate solution. The gel pieces were next reduced in 10 mM dithiothreitol and alkylated in 55 mM iodoacetamide. Overnight digestion was carried out with 12.5 ng/µL trypsin at 37 °C. Peptides were extracted from the gel pieces in 30 – 100 % acetonitrile solution, acidified and stage tipped (3) before injecting into the mass spectrometer. Samples were analyzed on an Q Exactive mass spectrometer coupled to an Easy-nLC 1200 (Thermo Fisher Scientific). Chromatographic separation was performed using an in-house constructed pre-column (45 mm × 0.075 mm I.D) and analytical (300 mm × 0.075 mm I.D.) column set up packed with 3 µm Reprosil-Pur C18-AQ particles (Dr. Maisch GmbH). Peptides were injected onto the column at a flow rate of 3 µL/min and 600 bars. Peptides were subsequently eluted using a segmented gradient over 60 min. The mass spectrometer was operated on a data-dependent mode. Survey full-scans for the MS spectra were recorded between 400 and 1600 Thompson at a resolution of 60,000 with a target value of 1e6 charges in the Orbitrap mass analyzer. The top 10 most intense peaks from the survey scans were selected for fragmentation with higher-energy collisional dissociation (HCD). Dynamic exclusion was set for 10 s. Raw data were processed with the MaxQuant software 1.6.3.4 (4). Database search was performed against a target-decoy database of *S. pneumoniae R6* downloaded from UniProt (UP000000586), containing 2031 protein entries (including 246 common laboratory contaminants). Endoprotease Trypsin/P was selected as the endoprotease, along with maximum missed cleavage of two. Oxidation on Methionine, acetylation of protein N-terminus and phosphorylation on Serine, Threonine and Tyrosine were selected under variable modifications. Carbamidomethylation on Cysteine was set as a fixed modification. A false discovery rate of 1% was applied at the peptide and protein level. All other parameters were maintained as default. Alternatively, we also performed NanoLC/nanospray/tandem mass

spectrometry experiments on a Q-STAR XL instrument (QqTOF) (Applied Biosystems, Courtaboeuf, France) equipped with a nanospray source using a distal coated silica-tip emitter (FS150-20-10-D-20, New Objective) set at 2300 V. By means of information-dependent acquisition mode, peptide ions within a m/z 400–2000 survey scan mass range were analyzed for subsequent fragmentation (three precursors). Double to quadruple charged ions exceeding a threshold of 10 counts were selected for MS/MS analyses. Screening for phosphorylated peptides was achieved by the paragon method from the Protein-Pilot data base-searching software (version 2.0, Applied Biosystems). The LC part of the analytical system consisted of an LC-Packings nano-LC (Dionex, Voisins Le Bretonneux, France). Chromatographic separation of peptides was obtained in a C18 PepMap micro-precolumn (5 µm; 100 Å; 300 µm x 5 mm; Dionex) and a C18 PepMap nano-column (3 µm; 100 Å; 75 µm x150 mm; Dionex). After injection (1-µl injection volume, pick-up mode, in a 20-µl injection loop), samples were adsorbed and desalted on the pre-column with a H_2O/CH_3CN/ trifluoroacetic acid (98:2:0.05; v/v/v) solvent mixture for 3 min at 25 µl/min flow rate. The peptide separation was developed using a linear 60-min gradient from 0 to 60 % B, where solvent A was 0.1% HCOOH in H_2O/CH_3CN (95/5) and solvent B was 0.1% HCOOH in H_2O/CH_3CN (20/80) at 200 nL/min flow rate.

To identify the protein partners of GFP-StkP-7TA and GFP-StkP-7TE, 50 µL of of GFP-StkP-7TA and GFP-StkP-7TE co-immunoprecipitation samples were mixed with 50 µL of Lyse buffer provided in the PreOmics iST Kit (PreOmics GmbH; Martinsried, Germany). The samples were reduced and alkylated by incubation at 60°C for 10 minutes with continuous shaking at 1000 rpm. The samples were then digested for 3 hours at 37°C and processed according to the manufacturer's protocol. The samples were analyzed using an Ultimate 3000 nano-RSLC (Thermo Scientific, San Jose, California) coupled online with a Q Exactive HF

mass spectrometer via a nano-electrospray ionization source (Thermo Scientific, San Jose, California). Four hundred and fifty nanograms of each peptide mixture were loaded onto a PepMap NEO C18 trap-column (300 µm ID × 5 mm, 5 µm, Thermo Fisher Scientific) for 3.0 minutes at a flow rate of 20 µL/min with 2% ACN, 0.05% TFA in H2O. They were then separated on a C18 Acclaim PepMap100 nano-column (50 cm × 75 µm i.d, 2 µm, 100 Å, Thermo Scientific) with a 100-minute linear gradient from 3.2% to 20% buffer B (A: 0.1% FA in H2O, B: 0.1% FA in ACN), followed by 20% to 32% buffer B in 20 minutes, 32% to 90% buffer B in 2 minutes, held for 10 minutes, and returned to initial conditions in 2 minutes for 13 minutes. The total run time was 150 minutes at a flow rate of 300 nL/min. The oven temperature was maintained at 40°C.

Samples were analyzed using a TOP15 HCD method. MS data were acquired in a data-dependent strategy, selecting the 15 most abundant precursor ions in the survey scan (350-1650 Th) for fragmentation. The resolution of the survey scan was 120,000 at m/z 200 Th. The Ion Target Value for the survey scans in the Orbitrap and the MS2 mode were set to 3E6 and 1E5, respectively, with a maximum injection time of 60 ms for both scan modes. HCD MS/MS spectra acquisition parameters were as follows: collision energy = 27; isolation width of 1.4 m/z; precursors with unknown charge state or a charge state of 1 were excluded. Peptides selected for MS/MS acquisition were placed in an exclusion list for 20 seconds using dynamic exclusion mode to limit duplicate spectra. The spray voltage was set to 1800 V, and the ion transfer tube was maintained at 250°C._Proteins were identified by database searching using Sequest HT with Proteome Discoverer 2.5 software (Thermo Scientific) against the *Streptococcus pneumoniae* R6 Uniprot database (2023-09 release, 2031 sequences), sequences of StkP-7TE and StkP-7TA, and a contaminant database. Precursor and fragment mass tolerances were set at 10 ppm and 0.02 Da, respectively, with up to two missed cleavages allowed. Oxidation (M), Phosphorylation (S, T, Y), and Acetylation (Protein N-terminus) were

set as variable modifications, and Carbamidomethylation (C) as a fixed modification. Full trypsin was selected as the digestion enzyme parameter. Peptide and protein validation was performed by Percolator, with a false discovery rate of 1% set for both peptides and proteins. Protein quantification was done using Label-Free Quantification (LFQ) approach, with LFQ abundance values normalized to the total peptide amount. Protein quantitation was performed using the precursor ions quantifier node in Proteome Discoverer 2.5 software, based on pairwise ratios and hypothesis t-tests. Proteins were considered differentially expressed between the two conditions when fold change (FC) > 1.8 or FC < 0.55 and p-value (pv) < 0.05.

REFERENCES

1. Fleurie A, Cluzel C, Guiral S, Freton C, Galisson F, Zanella-Cleon I, Di Guilmi A-M, Grangeasse C. 2012. Mutational dissection of the S/T-kinase StkP reveals crucial roles in cell division of Streptococcus pneumoniae. Mol Microbiol 83:746–758. https://doi.org/10.1111/j.1365-2958.2011.07962.x

2. Fleurie A, Manuse S, Zhao C, Campo N, Cluzel C, Lavergne J-P, Freton C, Combet C, Guiral S, Soufi B, Macek B, Kuru E, VanNieuwenhze MS, Brun YV, Di Guilmi A-M, Claverys J-P, Galinier A, Grangeasse C. 2014. Interplay of the serine/threonine-kinase StkP and the paralogs DivIVA and GpsB in pneumococcal cell elongation and division. PLoS Genet 10:e1004275. https://doi.org/10.1371/journal.pgen.1004275

3. Ishihama Y, Rappsilber J, Mann M. 2006. Modular Stop and Go Extraction Tips with Stacked Disks for Parallel and Multidimensional Peptide Fractionation in Proteomics. J Proteome Res 5:988–994. https://doi.org/10.1021/pr050385q

4. Cox J, Matic I, Hilger M, Nagaraj N, Selbach M, Olsen JV, Mann M. 2009. A practical guide to the MaxQuant computational platform for SILAC-based quantitative proteomics. Nat Protoc 4:698–705. https://doi.org/10.1038/nprot.2009.36
